# Disruption of *hmgA* by DNA Duplication is Responsible for Hyperpigmentation in a *Vibrio anguillarum* Strain

**DOI:** 10.1038/s41598-019-51126-8

**Published:** 2019-10-10

**Authors:** Veronica Batallones, Jennifer Fernandez, Brett Farthing, Jordan Shoemaker, Keizen Li Qian, Kimberly Phan, Eric Fung, Ashley Rivera, Kevin Van, Francesca de la Cruz, Alexandra J. Ferreri, Krystle Burinski, Jackie Zhang, Vicente Lizarraga, Kevin Doan, Kenneth Rocha, German Traglia, Maria S. Ramirez, Marcelo E. Tolmasky

**Affiliations:** Center for Applied Biotechnology Studies, Department of Biological Science, California State University Fullerton, Fullerton, CA USA

**Keywords:** Genetics, Microbial genetics

## Abstract

*Vibrio anguillarum* 531A, isolated from a diseased fish in the Atlantic Ocean, is a mixture composed of about 95 and 5% of highly pigmented cells (strain 531Ad) and cells with normal levels of pigmentation (strain 531Ac), respectively. Analysis of the *V*. *anguillarum* 531Ad DNA region encompassing genes involved in the tyrosine metabolism showed a 410-bp duplication within the *hmgA* gene that results in a frameshift and early termination of translation of the homogentisate 1,2-dioxygenase. We hypothesized that this mutation results in accumulation of homogentisate that is oxidized and polymerized to produce pyomelanin. Introduction in *E*. *coli* of recombinant clones carrying the *V*. *anguillarum hppD* (4-hydroxyphenylpyruvate-dioxygenase), and a mutated *hmgA* produced brown colored colonies. Complementation with a recombinant clone harboring *hmgA* restored the original color to the colonies confirming that in the absence of homogentisate 1,2-dioxygenase the intermediary in tyrosine catabolism homogentisate accumulates and undergoes nonenzymatic oxidation and polymerization resulting in high amounts of the brown pigment. Whole-genome sequence analysis showed that *V*. *anguillarum* 531 Ac and 531Ad differ in the *hmgA* gene mutation and 23 mutations, most of which locate to intergenic regions and insertion sequences.

## Introduction

*Vibrio anguillarum* is the causative agent of *vibriosis*, a devastating disease characterized by terminal hemorrhagic septicemia that affects numerous fresh and salt-water fish species^1,2^. Among the hosts that *V*. *anguillarum* infects are species of enormous economic importance such as salmon, turbot, rainbow trout, sea bass, sea bream, cod, eel, and ayu^1^. Although never recognized as a human pathogen, one case of fatal infection in an immunocompromised patient has been documented^3^. Studies on the molecular mechanisms of pathogenicity in this bacterium led to the identification of various virulence factors^1,4,5^ such as motility^6–9^, adhesion^9^, the lipopolysaccharide^1^, outer membrane proteins^1^, DNase, protease, and hemolysin activities^4,10–13^, as well as efficient iron uptake systems^14–17^. *V*. *anguillarum* also possesses quorum sensing systems involved in regulation of expression of virulence factors^18,19^. The pJM1-type plasmid-mediated anguibactin iron-uptake system is one of the best characterized virulence factors present in numerous serotype O1 *V*. *anguillarum* strains^14,15,20,21^. It consists of the low-molecular-weight siderophore anguibactin and a receptor complex that facilitates transport of ferric-anguibactin across the membranes^22,23^. The vast majority of the pJM1-type plasmids include genes that code for enzymes that participate in the synthesis of anguibactin, outer membrane and periplasmic components of the ferric-anguibactin receptor complex, and regulatory elements^24–27^. Studies on *V*. *anguillarum* 531A, a strain isolated from a diseased fish on the Atlantic coast, showed that it carries a pJM1-like plasmid, pJHC1, and can grow in the presence of higher concentrations of iron chelators when compared to other strains like the well-studied strain 775^28,29^. Another unique and intriguing property of *V*. *anguillarum* 531A is that it secretes very high amounts of a brown pigment. We aimed to understand the genetics and biochemistry of this property. In this work, we report that *V*.*anguillarum* 531A is a mix of two variants, one of them producing dark brown colonies due to production of an ochronotic-like pigment that is correlated with a DNA duplication that disrupts the *hmgA* gene. The lack of a functional homogentisate 1,2-dioxygenase leads to accumulation of homogentisate in the tyrosine degradation pathway. Numerous reports described bacteria that produce the ochronotic pigment pyomelanin when homogentisate accumulates and undergoes oxidation and polymerization^30–38^.

## Results

**Figure 1:**
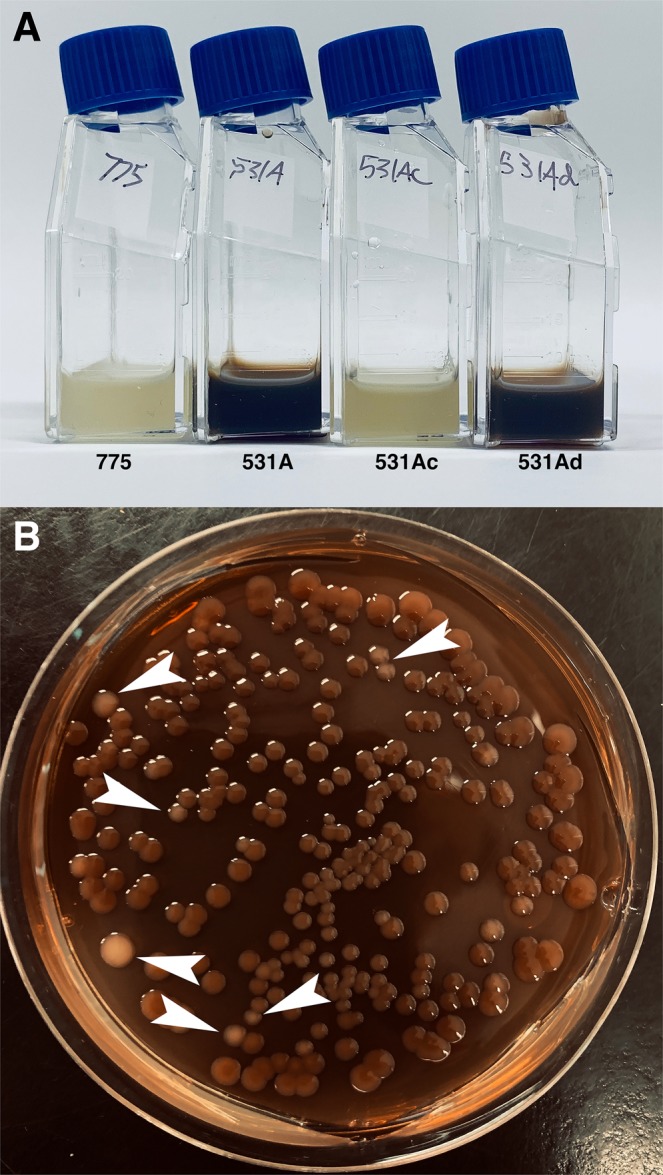
Pigment production in *V*. *anguillarum* strains. (**A**) Cultures were carried out in TSBS at 25 °C for 72 h with shaking. (**B**) *V*. *anguillarum* 531A was plated in TSAS and incubated at 25 °C for 48 h. The white arrowheads show some of the clear colonies. Variants 531Ac and 531Ad were isolated from a similar plate.

*V*. *anguillarum* 531A, a strain isolated from diseased *Oncorhynchus kisutch* in the Atlantic Ocean, carries the pJM1-type plasmid pJHC1 and produces higher levels of anguibactin than the strain 775^28,29,39^. While performing assays to characterize *V*. *anguillarum* 531A, we noticed that, unlike 775 and all other *V*. *anguillarum* strains in our collection, it produces a dark brown pigment (Fig. 1A). Another observation was that upon plating, the isolate produced two kinds of colonies, about 95% of them were dark and the rest with the usual clear color (Fig. 1B). A representative of each of the colonies was isolated, and the new strains were named 531Ad (dark) and 531Ac (clear). These strains conserved their pigmentation characteristics when they were subsequently cultured in TSBS or TSAS (Fig. 1A).

**Figure 2:**
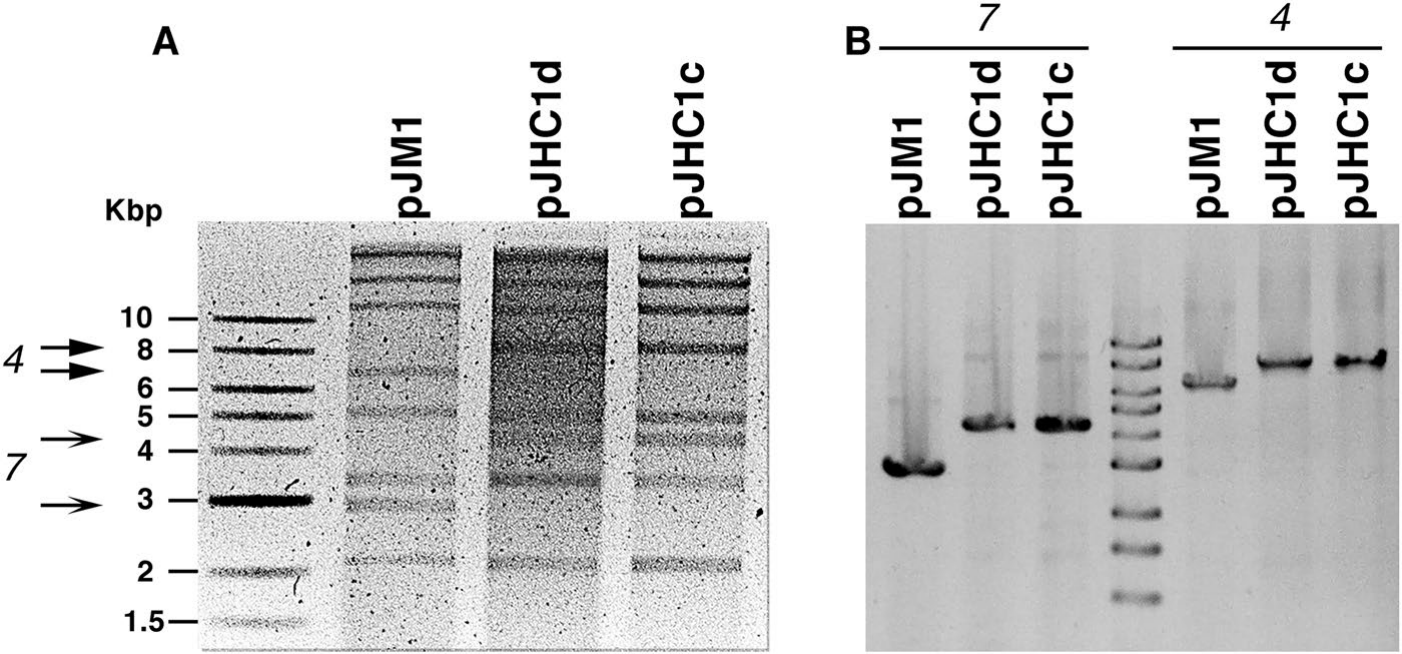
Restriction endonuclease digestion of *V*. *anguillarum* plasmids. Ethidium bromide-stained 0.7% agarose gel electrophoresis of *Bam*HI digestions of plasmids pJM1, pJHJC1d, and pJHC1c. Original pictures of both gels are shown in the Supplementary Information.

To determine if *V*. *anguillarum* 531Ac and 531Ad were two independent strains or one derives from the other we did whole-genome sequencing. The draft sequences of strains 531AC and 531AD had a total of 2,039,599 and 2.204.992 high-quality paired-end reads, respectively; 99.9% of the reads were mapped, resulting in mean nucleotide coverage of 30X for both genomes. The corrected reads showed an average length of 128 (531Ac) and 123 (531Ad) bp and there were 119 (531Ac) and 124 (531Ad) contigs larger than 500 bp. The draft genomes have an N_50_ contig size of 96,636 (531Ac) and 100,331 (531Ad) bp with a maximum contig length of 347,928 (531Ac) and 321,926 (531Ad). The GC content (%) of the genomes was determined as 44.3%, a value consistent with previous measurements using other *V*. *anguillarum* strains^40^. The genomic differences between both strains were evaluated by performing genome comparisons. Two-way average nucleotide identity (ANI) calculation and the tetra nucleotide correlation analysis among both isolates showed a 99,9% value and a score 1.0, respectively. Also, tetra nucleotide correlation against all the database of JSpeciesWS, showed the highest score against *V*. *anguillarum* 775. These results strongly suggest that *V*. *anguillarum* 531Ac and 531Ad could be considered to be the essentially same strain with a small number of nucleotide differences. To validate these results, a core-genome phylogeny of *V*. *anguillarum* 531Ac and 531Ad and *V*. *anguillarum* genomes from GenBank. The core-genome contains 1538 genes and the pan-genome contains 13,038 genes. The phylogenetic relation among the *V*. *anguillarum* 531Ac and 531Ad genomes as determined by core-genome phylogeny analysis showed that the pairwise distance is 0.000344655 substitution per 100 bp, which indicates the presence of a few SNPs between both isolates (Supplementary Fig. S1 and Table S1). Mutation prediction among both isolates showed that besides the duplication of of the *hmgA* gene described below, there are 23 mutations among the *V*. *anguillarum* 531 Ac and 531Ad genomes. While most mutations were found within intergenic regions and insertion sequence (ISVa5 and ISVa2) (Supplementary Table S2), one of them occurred within the TyrP (tyrosine-specific transport protein) coding sequence. This mutation resulted in a Q349P amino acid change (Supplementary Table S2) within the transmembrane domain. However, prediction of transmembrane helices using the TMHMM software showed that the mutation does not induce significant structural differences. As a consequence, it is most probable that the one amino acid difference does not affect tyrosine uptake. Future research will permit to confirm this observation. A final confirmation of the identity between *V*. *anguillarum* 531Ac and 531Ad was carried out by restriction analysis of their plasmid content. *V*. *anguillarum* strains usually harbor pJM1-type plasmids whose DNA sequences slightly differ^14,28,41,42^. Therefore, identical restriction digestion patterns of both pJM1-type plasmids would be a strong indication that one of the strains derives from the other. The plasmid originally isolated from *V*. *anguillarum* 531A was called pJHC1, then the plasmids isolated from the variants 531Ac and 531Ad were called pJHC1c and pJHC1d. Figure 2A shows a comparison of the products of *Bam*HI restriction digestion of pJHC1c and pJHC1d, as well as the prototype plasmid pJM1. This figure shows that both strains, 531Ac and 531Ad, shared the same signature pJM1-type plasmid, the *Bam*HI fragments 4 and 7 showed identical molecular size in pJHC1c and pJHC1d, and were larger than those from pJM1. The correspondence between the fragments was confirmed by PCR using as primers oligonucleotides at the edges of the *Bam*HI fragments 4 and 7 based on the pJM1 nucleotide sequence (Fig. 2B). These results agree with the genomic analyses and indicates that both variants have a common origin.

**Figure 3:**
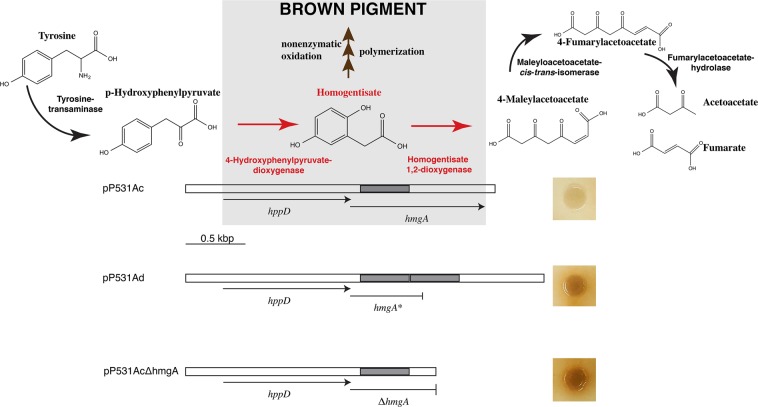
Pathway for tyrosine metabolism and production of the brown pigment. The top portion of the figure shows the tyrosine catabolic pathway and the key enzymes for formation of the brown pigment are shown within the gray box. The box also includes the region of DNA that includes the *hppD* and *hmgA* genes. The lower portion of the figure shows diagrams of the fragments and genes included in all three recombinant plasmids. The grayed region is directly repeated in the 531Ad strain; hmgA* indicates that the 410-bp tandem duplication (highlighted in gray) in this gene produces a sequence that codes for a truncated homogentisate 1,2-dioxygenase (Supplementary Fig. S2). The phenotypes produced by each recombinant plasmid in *E*. *coli* cultured in LB agar are shown to the right of the genetic maps.

**Figure 4:**
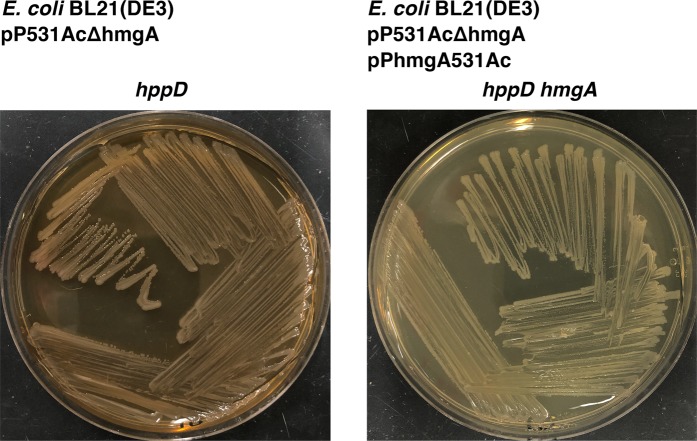
h*mgA* gene complementation. *E*. *coli* BL21(DE3) (pP531AcΔhmgA) and *E*. *coli* BL21(DE3) (pP531AcΔhmgA, pPhmgA531Ac) were plated on Trypticase soy agar with the addition of 1% (w/v) NaCl.

Production of the ochronotic type pigment pyomelanin is caused by the accumulation of homogentisate formed by the action of 4-hydroxyphenylpyruvate dioxygenase, coded for by the *hppD* gene, on 4-hydroxyphenylpyruvic acid in the tyrosine metabolic pathway (Fig. 3)^33,34,43,44^. Accumulation of homogentisate can occur when homogentisate 1,2-dioxygenase, the enzyme encoded by *hmgA* that metabolizes this compound to 4-maleylacetoacetate, is not present (Fig. 3). The homogentisate is oxidized and polymerized to produce pyomelanin^30,34^. Figure 3 shows the tyrosine metabolic pathway underscoring the steps relevant to formation of pyomelanin. To determine if this pathway is responsible for the hyperproduction of the brown pigment in *V*. *anguillarum* 531Ad, we cloned the chromosome I region encompassing the *hppD* and *hmgA* genes from strains 531Ac and 531Ad. Introduction of the recombinant clones pP531Ac and pP531Ad into *E*. *coli* resulted in clear and dark colonies, respectively (Fig. 3). Inspection of the nucleotide sequences of both regions cloned showed that there was a 410-bp tandem repeat within the nucleotide sequence of the *V*. *anguillarum* 531Ad *hmgA* gene (hmgA*) that results in a frameshift and truncation of the product of translation. This modification leaves 156 out of a total of 375 amino acids in the complete protein (Fig. 3 and Supplementary Fig. S2). To confirm that the lack of a functional homogentisate 1,2-dioxygenase was responsible for the high production of pigment, the region cloned in pP531Ac was used as template to generate a DNA fragment that includes the complete *hppD* and only a portion of the *hmgA* gene. This fragment was used to generate the recombinant clone pP531AcΔhmgA (Fig. 3 and Supplementary Fig. S2). As expected, *E*. *coli* cells carrying this recombinant plasmid produced highly pigmented colonies (Fig. 3). Next, a recombinant clone harboring the *hmgA* gene under the control of the T7 promoter was generated inserting the appropriate DNA segment from strain 531Ac into pACYC177, a vector compatible with pCR-Blunt II-TOPO. This plasmid, pPhmgA531Ac, was introduced into *E*. *coli* BL21(DE3) already harboring pP531AcΔhmgA, which produces brown colonies (Fig. 4). The transformant strain, *E*. *coli* BL21(DE3) (pP531AcΔhmgA, pPhmgA531Ac), produced clear colonies further confirming that lack of a functional *hmgA* gene is a direct cause of production of the brown pigment (Fig. 4). The experiments described in this paragraph led us to conclude that *V*. *anguillarum* 531Ad produces high amounts of pyomelanin due to the lack of a functional homogentisate 1,2-dioxygenase. Inspection of all 60 complete *V*. *anguillarum* genomes in GenBank indicated that all of them include a gene coding for a full homogentisate 1,2-dioxygenase. Therefore, 531Ad is the only *V*. *anguillarum* strain described to date that harbors a disrupted *hmgA* gene.

It had been observed that *V*. *anguillarum* 531A can grow at higher concentrations of iron chelators when compared to *V*. *anguillarum* 775 due to higher production of anguibactin^28,29^. Since *V*. *anguillarum* 531A is a mix of two variants, 531Ac and 531Ad, it is possible that only one of them possesses the ability to grow at higher concentrations of iron chelators. The minimal inhibitory concentrations of EDDHA for both variants were 20 μM while the value for strain 775 was 10 μM. This result indicated that the presence or absence of an intact copy of the *hmgA* gene does not impact the iron proficiency of *V*. *anguillarum* in the experimental conditions.

Coculturing assays were used to compare the fitness of strains 531Ad and 531Ac to determine if the ability to produce high amounts of pyomelanin in high quantities was advantageous when growing in TSBS culture medium. A 1:1 mixture of the strains was cultured, and the relative frequencies were determined every 20 generations. Up to 60 generations, the ratio 531Ad/531Ac was approximately 1 indicating that both strains were equally fit when growing in TSBS.

**Figure 5:**
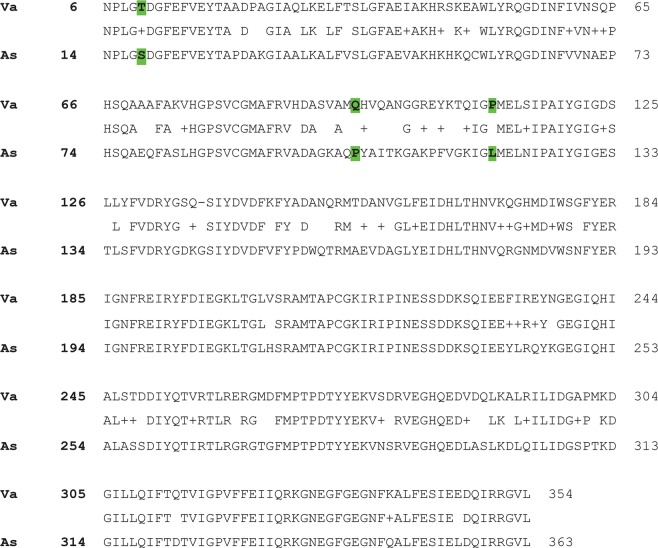
4-Hydroxyphenylpyruvate-dioxygenase amino acid sequences comparison. Alignment of the amino acid sequences of the 4-hydroxyphenylpyruvate-dioxygenase proteins from *V*. *anguillarum* (Va) and *A*. *salmonicida* (As) (accession number MH909233.1). Amino acids involved in thermolability are highlighted in green.

Since a recent report described a variant of the *Aeromonas salmonicida* 4-hydroxyphenylpyruvate dioxygenase that is thermolabile due to the amino acids S18, P103, and L119, which in thermostable variants are mostly T18, Q103, and P119^37^, we identified these important amino acids within the *V*. *anguillarum* version of the enzyme. A comparison of both sequences showed that except for a short stretch at the N-terminus, the amino acid sequences shared 76% identity and 85% similarity (Fig. 5). Furthermore, the amino acids at the key positions for thermolability in the *V*. *anguillarum* enzyme were T, Q, and P as it is the case of those that are thermostable (Fig. 5).

## Discussion

The production of the ochronotic pigment pyomelanin has been reported in numerous organisms including humans, where the accumulation of homogentisate produces the syndrome known as ochronosis or alkaptonuria^45,46^. Numerous bacteria have been identified as producers of a pyomelanin-type pigment, which occurs through the metabolic pathway of tyrosine degradation. Homogentisate is produced by the action of 4-hydroxyphenylpyruvate dioxygenase on 4-hydroxyphenylpyruvic acid. When this metabolite accumulates, it undergoes oxidation and polymerization producing the brown pigment pyomelanin. This process has been identified as the cause behind the production of the pigment in several bacteria such as *V*. *anguillarum*^33^, *V*. *cholerae*^38^, *A*. *salmonicida*^37^, *Legionella pneumophila*^31^, *Shewanella colwelliana*^32^, *Burkholderia cepacia*^35^, *B*. *cenocepacia*^36^, *Streptomyces avermitilis*^34^, *Ralstonia solanacearum*^30^. Previous studies identified VanT and RpoS as positive regulators of pyomelanin production by enhancing expression of 4-hydroxyphenylpyruvate dioxygenase in *V*. *anguillarum* cultures entering stationary phase^33,44^. However, based on the published information^33,44^, these levels of pigment production must be much lower than those observed in strain 531Ad, in which the lack of homogentisate 1,2-dioxygenase activity leads to accumulation of the pyomelanin precursor and production of unusually high amounts of the pigment. The results obtained by searching the GenBank database indicating that all sequenced *V*. *anguillarum* strains show a complete *hmgA* gene confirms that the mechanism of production of pyomelanin in strain 531Ad is, to our best knowledge, exceptional among this species. A search for this characteristic, i.e., higher pigmentation, in other bacteria showed that a subset of *Burkholderia cepacia* strains includes a deficient *hmgA* gene. In this case, rather than the truncation, the variant of the *hmgA* gene codes for a protein that has an amino acid substitution that is detrimental for enzymatic activity^35^.

Since we did not know the basis of the appearance of two variants in the sample kept at −80 °C in the laboratory collection, a battery of experiments was carried out to determine of both *V*. *anguillarum* 531Ac and 531Ad have a common origin and also if there is a fitness difference in regular laboratory growth conditions. Whole-genome sequencing and plasmid restriction enzyme digestion analyses showed that both variants are essentially the same strain with the exception of the *hmgA* gene and other minor mutations. Furthermore, comparison of their iron uptake proficiency also failed to show any difference. Of course, it is possible that the production of pyomelanin is advantageous in particular conditions not tested in this work. For example, it was recently found in comparisons of different *Klebsiella pneumoniae* strains, which showed that a dominant *Klebsiella pneumoniae* strain in the host does not show enhanced fitness in the mixed growth assay^47^. Several studies found pyomelanin functions that could enhance fitness in bacteria. Examples of these functions are protection against oxidative stress^30,36,48^ or enhancement of bacterial virulence^35,38^. A *V*. *cholerae* mutant obtained by mini-Tn5 insertion within *hmgA* overproduced pyomelanin, expressed more toxin-coregulated pilus and cholera toxin, and showed enhanced colonization of intestines in an infant mice model of infection^38^. On the other hand, other reports show a reduction in virulence of bacteria that develop the capability to produce the pigment^49^. The information available seems to indicate that high production of pyomelanin is correlated with pleiotropic changes that may differ among bacteria. Future experiments will clarify our understanding of the possible role of pyomelanin in high quantities in the physiology and virulence of *V*. *anguillarum*.

## Methods

### Bacterial strains, plasmids, primers, and media

The relevant characteristics of bacterial strains and plasmids used in this work are described in Table 1. *V*. *anguillarum* 531A was isolated from a diseased fish in the coast of Maine^28,29,39^. *V*. *anguillarum* 775 is a prototype serotype O1 strain; it was used for comparison of growth under iron deficiency conditions and as a source of plasmid pJM1^22^. *Escherichia coli* TOP10 (Life Technologies) and BL21(DE3)^50^ were used as hosts of recombinant clones. Plasmids pCR-Blunt II-TOPO (Life Technologies) and pACYC177^51^ were used as cloning vehicles. *V*. *anguillarum* strains were cultured at 25 °C in Trypticase soy broth with the addition of 1% (w/v) NaCl (TSBS), and for cultures in solid medium the medium was supplemented with 2% agar (TSAS)^42^. *E*. *coli* strains were cultured at 37 °C in LB broth or LB agar (2%). Oligonucleotides to be used as primers or for DNA sequencing were purchased from Integrated DNA Technologies or Bio-Synthesis Inc.

**Table 1:** Bacterial strains and plasmids used in this study.

Bacteria or plasmids	Relevant characteristics or genotype	Source or reference
**Bacteria**		
*V*. *anguillarum* 531A	Isolate from an infected fish. Carries pJHC1, a pJM1-like plasmid	Tolmasky *et al*.^29^
*V*. *anguillarum* 531Ad	High pigment producer	This work
*V*. *anguillarum* 531Ac	Low, or no, pigment producer	This work
*E*. *coli* TOP10	F^−^ *mcrA* Δ(*mrr-hsd*RMS-*mcr*BC) Φ80*lac*ZΔM15 Δ*lac*X74 *rec*A1 *ara*D139 Δ(*ara-leu*)7697 *galU galK rpsL* (StrR) *end*A1 *nupG*	Life Technologies
*E*. *coli* BL21(DE3)	F^−^ *ompT hsdSB* (*rB*^−^*mB*^−^) *gal dcm* (DE3)	Studier & Moffatt, 1986^50^
**Plasmids**		
pCR-Blunt II-TOPO	Cloning vector KAN^r^ ZEO^r^	Life Technologies
pACYC177	Cloning vector KAN^r^ AMP^r^	Rose, 1988^51^
pP531Ac	*hppD*-*hmgA* fragment from strain 531Ac cloned into pCR-Blunt II-TOPO	This work
pP531Ad	*hppD*-hmgA* fragment from strain 531Ad cloned into pCR-Blunt II-TOPO	This work
pP531AcΔhmgA	*hppD*-Δ*hmgA* fragment from strain 531Ac cloned into pCR-Blunt II-TOPO	This work
pPhmgA531Ac	*hmgA* fragment from strain 531Ac cloned into pACYC177	This work

### General procedures

Plasmid DNA isolation and PCR reactions were carried out using the QIAspin miniprep (QIAGEN) and the Invitrogen Platinum SuperFi PCR Master Mix (ThermoFisher Scientific) kits, respectively. Primers used to amplify DNA fragments are listed in Table 2. Restriction endonuclease and T4 DNA ligase treatments were performed according to the supplier’s recommendations (New England Biolabs). Transformation of competent *E*. *coli* strains was carried out as described by the supplier (Life Technologies). Briefly, 50 μl of the competent cells suspension were thawed on ice before adding the DNA sample. The cells were then incubated for 30 minutes on ice, followed by a heat shock at 42 °C for 30 seconds. Then 250 μl of LB broth were added, and the cells were incubated for 1 hour at 37 °C with shaking. The cells were then plated on LB agar containing 1 mg/ml ampicillin. Amino acid sequence comparisons were carried out using the BLAST tool at the National Center for Biotechnology information^52^.

**Table 2:** Primers.

Primer name	Sequence	Coordinates^a^
F^a^	AGGCAGTGTCGGTTTTTAATG	1–21
R^a^	TAACCCATCTCTTGTGCCGT	2586–2567
R2^a^	CCACGGAAAGATCACCGTGCC	2093–2073
F*hmgA*T7^a,b^	*ATCGAT*GCGAAATTAATACGACTCACTATAGGGGAGATCATTCAACGCAAAGGCAATC	1288–1311
R*hmgA*T7^a,b^	*AAGCTT*AACCCATCTCTTGTGCCGTTTTTTAG	2586–2560
BamHI4F^c^	GAATAGATCAGCTGCTTTGGGATCC	34731–34755
BamHI4R^c^	ATCCCAATGTTTCAATGTACGAGGATCC	41395–41368
BamHI7F^c^	GATCCTCTTTTCGTTCAGCACTGG	55321–55344
BamHI7R^c^	GGCGTGGTGTGCTTTCGGATC	58207–58187

^a^Coordinates according to the *Vibrio anguillarum* 775 chromosome I sequence, accession number CP002284, region: 1733068.1737000.

^b^Double underlined sequence is the T7 promoter. Single underlined and italics underlined are the *Cla*I and *Hin*dIII restriction sites, respectively.

^c^Coordinates according to the *Vibrio anguillarum* 775 pJM1 plasmid, accession number AY312585.

### Determination of minimal inhibitory concentration (MIC) of ethylenediamine di(*o*-hydroxyphenyl) acetic acid (EDDHA)

MIC of the synthetic iron chelator of the iron chelator EDDHA was assessed culturing the strains in M9 minimal medium containing incremental EDDHA concentrations up to 40 μM^22,28^.

### Fitness assays

Fitness of *V*. *anguillarum* strains 531Ac and 531Ad were compared coculturing a 1:1 mixture of the strains and determining their ratio every 20 generations as described previously^53,54^.

### Pigment production

*V*. *anguillarum* strains were inoculated in TSBS or plated on solid media and cultured at 25 °C for 36 h. *E*. *coli* strains were plated on LB agar and incubated for 36 h at 37 °C. The production of pigment was readily visible.

### Genome sequencing and bioinformatic analysis

Genomic DNA was extracted using the Master Pure DNA purification kit following the manufacturer’s instructions (Epicentre, Madison, WI, USA). Libraries were generated using the Illumina Nextera kit (Illumina Inc., San Diego, CA) as described by Baym *et al*.^55^, and sequenced with the Illumina NextSeq500 equipment at the Microbial Genome Sequencing Center (Pittsburgh, PA). De novo assembly was performed with SPADES assembler, version 3.3.1^56^. The annotation was performed using the RAST server^57^ and the predictions were confirmed using the BLAST software^52^. The quality of the sequences and assembly were evaluated using FASTQC software (https://www.bioinformatics.babraham.ac.uk/projects/fastqc/) and the QUAST software^58^, respectively. The Average Nucleotide Identity and pairwise tetra nucleotide correlation were performed using JSpeciesWS online service^59^. The mutation prediction between both isolates was performed using breseq^60^. The core genome prediction was carried out using the Roary software^61^ including seventy *V*. *anguillarum* genomes from GenBank. The core-genome phylogeny was performed using RaxML^62^ and substitution model was predicted with JModelTest2^63^. The default Bootstrap test was used to evaluate branch supports. The tree representation was done using FigTree (https://github.com/rambaut/figtree/). The transmembrane domain prediction was done using the TMHMM software (http://www.cbs.dtu.dk/services/TMHMM/).

## Supplementary information

Supplementary Information

## Data Availability

Published accession numbers of sequences used in this study are included in the article. The nucleotide sequences of specific regions from strains 531Ac and 531Ad have been provided GenBank accession numbers MK791314 and MK791315. Whole genome sequences of *V*. *anguillarum* 531Ac and 531Ad provided GenBank accession numbers VSLE00000000 and VSLF00000000, respectively. Bacterial strains are available upon request.
