# Supplementary material for: Disruption of hmgA by DNA Duplication is Responsible for Hyperpigmentation in a Vibrio anguillarum Strain
Source: Sci Rep. 2019 Oct 10;9:14589. doi: 10.1038/s41598-019-51126-8 (PMC6787238; doi:10.1038/s41598-019-51126-8)
Supplement: Supplementary file 1 — Supplementary Information [file 41598_2019_51126_MOESM1_ESM.pdf]

Supplementary Information

Disruption of *hmgA* by DNA Duplication is Responsible for Hyperpigmentation in a *Vibrio anguillarum* Strain

Veronica Batallones, Jennifer Fernandez, Brett Farthing, Jordan Shoemaker, Keizen Li Qian, Kimberly Phan, Eric Fung, Ashley Rivera, Kevin Van, Francesca de la Cruz, Alexandra J. Ferreri, Krystle Burinski, Jackie Zhang, Vicente Lizarraga, Kevin Doan, Kenneth Rocha, German Traglia, Maria S. Ramirez, and Marcelo E. Tolmasky*

Center for Applied Biotechnology Studies, Department of Biological Science, California State University Fullerton, Fullerton, CA, USA

Supplementary Fig S1

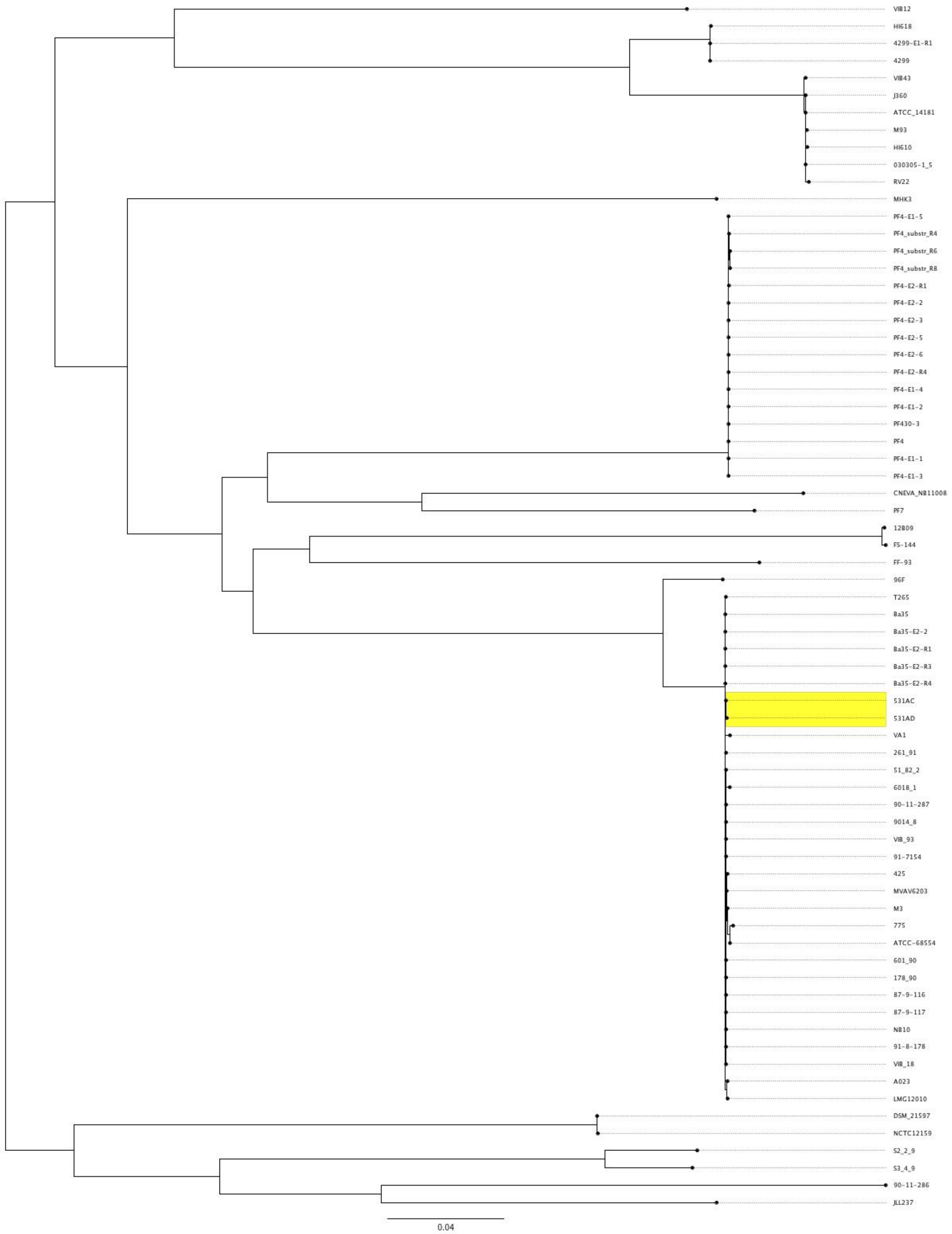

Supplementary Figure S1. Core-genome phylogeny representation of *Vibrio anguillarum*. The locations of *V. anguillarum* 531Ac and 531Ad in the tree are highlighted in yellow. A 58109 bp stretch was aligned of 1538 core-genes from 70 genomes of *V. anguillarum* using the ClustalX2 software. General Time Reversible substitution model was predicted using the JModelTest2 software. Phylogenetic analysis was performed using the Maximum Likelihood method.

Supplementary Fig S2

531Ac

```
     F
   1 AGGCAGTGTCGGTTTTTAATGAATGTAGCTAAAATGTTACGCCGTGCTATTTATCTTACT 60

  61 GAGCAACTGGTTATGATTGACTTGTGATTAACGTGGCCAATTAAGAAAAAGTTATTAATA 120

 121 TTCAGTCAAATAAAAACAAATCATCTCTGTGAAATTCGCTAATTTACTATCCTACTGCTC 180

 181 ATTAATAGATTTGTGTAACATTTTAATTACATTGACACTATTTGTTCTCCGCTAAACCGT 240

 241 TTTTCGTTATAGGGTTCATTTTTGCTGAAAATTACGCCATGTTCGCAATACTCAATACAA 300

 301 GCGTTAACAAAAAAGGAGCGTGAGATGACTTCAACATTTAACCCATTGGGAACAGACGGT 360
                             M  T  S  T  F  N  P  L  G  T  D  G

 361 TTTGAATTTGTGGAGTACACCGCGGCTGATCCCGCTGGAATCGCGCAGTTAAAAGAGCTG 420
     F  E  F  V  E  Y  T  A  A  D  P  A  G  I  A  Q  L  K  E  L

 421 TTTACTTCTCTTGGCTTTGCTGAGATAGCCAAACATCGCTCAAAAGAGGCGTGGTTATAT 480
     F  T  S  L  G  F  A  E  I  A  K  H  R  S  K  E  A  W  L  Y

 481 CGTCAAGGGGATATCAATTTTATCGTTAACTCTCAACCCCATAGCCAAGCTGCAGCGTTT 540
     R  Q  G  D  I  N  F  I  V  N  S  Q  P  H  S  Q  A  A  A  F

 541 GCCAAAGTGCATGGACCTTCAGTGTGTGGGATGGCGTTTCGCGTTCATGATGCATCTGTC 600
     A  K  V  H  G  P  S  V  C  G  M  A  F  R  V  H  D  A  S  V

 601 GCCATGCAGCATGTGCAGGCAAATGGCGGGCGAGAATACAAAACACAAATTGGCCCAATG 660
     A  M  Q  H  V  Q  A  N  G  G  R  E  Y  K  T  Q  I  G  P  M

 661 GAACTGAGCATTCCTGCGATTTATGGAATTGGCGATAGCTTGCTTTATTTTGTCGATCGT 720
     E  L  S  I  P  A  I  Y  G  I  G  D  S  L  L  Y  F  V  D  R

 721 TATGGTAGCCAGAGCATTTATGATGTGGATTTCAAGTTCTATGCCGATGCTAATCAACGA 780
     Y  G  S  Q  S  I  Y  D  V  D  F  K  F  Y  A  D  A  N  Q  R

 781 ATGACCGACGCCAATGTTGGCTTGTTCGAAATTGACCACTTAACGCATAACGTCAAACAA 840
     M  T  D  A  N  V  G  L  F  E  I  D  H  L  T  H  N  V  K  Q

 841 GGCCACATGGACATTTGGTCTGGCTTTTATGAGCGGATTGGTAACTTCCGTGAAATTCGC 900
     G  H  M  D  I  W  S  G  F  Y  E  R  I  G  N  F  R  E  I  R

 901 TATTTTGATATTGAAGGGAAGCTAACTGGGTTAGTTAGCCGCGCAATGACAGCGCCTTGC 960
     Y  F  D  I  E  G  K  L  T  G  L  V  S  R  A  M  T  A  P  C
```

```
 961 GGCAAAATTCGCATCCCAATCAATGAATCGTCTGACGATAAATCACAAATAGAAGAGTTT 1020
     G  K  I  R  I  P  I  N  E  S  S  D  D  K  S  Q  I  E  E  F

1021 ATTCGTGAATACAATGGTGAGGGGATCCAACACATCGCTTTATCCACTGATGATATCTAC 1080
     I  R  E  Y  N  G  E  G  I  Q  H  I  A  L  S  T  D  D  I  Y

1081 CAGACGGTACGTACTCTGCGCGAACGAGGGATGGATTTCATGCCAACACCGGATACTTAC 1140
     Q  T  V  R  T  L  R  E  R  G  M  D  F  M  P  T  P  D  T  Y

1141 TACGAAAAAGTCAGTGATCGAGTTGAAGGGCATCAAGAAGATGTTGATCAGTTAAAAGCG 1200
     Y  E  K  V  S  D  R  V  E  G  H  Q  E  D  V  D  Q  L  K  A

1201 CTGCGGATCCTGATTGATGGTGCACCGATGAAAGATGGAATTTTGCTGCAAATATTTACT 1260
     L  R  I  L  I  D  G  A  P  M  K  D  G  I  L  L  Q  I  F  T
ATCGATGCGAAATTAATACGACTCACTATAGGG

1261 CAAACCGTCATTGGCCCAGTCTTTTTTGAGATCATTCAACGCAAAGGCAATGAAGGGTTC 1320
     Q  T  V  I  G  P  V  F  F  E  I  I  Q  R  K  G  N  E  G  F

1321 GGGGAAGGTAATTTCAAAGCGTTATTTGAGTCGATTGAAGAAGATCAAATTCGACGAGGA 1380
     G  E  G  N  F  K  A  L  F  E  S  I  E  E  D  Q  I  R  R  G

1381 GTACTCAGCGATGCATAAATGGGTCACATTTCCTCATCGGGAAGGGGTATGTTCAAACCA 1440
     V  L  S  D  A  *
                M  H  K  W  V  T  F  P  H  R  E  G  V  C  S  N  Q

1441 AGCTCATGCCGACTTCCCGCCAGAGGCCATCTATGAAAGAGAAGCAGGACGCAGTGGCTT 1500
       A  H  A  D  F  P  P  E  A  I  Y  E  R  E  A  G  R  S  G  F

1501 TTTTGGCCCTGCGGCCCATTTCCATCACCAACATGCACCGACAGGGTGGAGTGAATGGCA 1560
       F  G  P  A  A  H  F  H  H  Q  H  A  P  T  G  W  S  E  W  Q

1561 AGGTGAGTTAAGGCCGAGAGCCTTTAATTTTAATTTGATCGAGCAAGCCAAACAAAGTTC 1620
       G  E  L  R  P  R  A  F  N  F  N  L  I  E  Q  A  K  Q  S  S

1621 ACCGTGGAGCGTGCCGCACCTATTGCATAATGCACAATGTAAAGTACGAGTATGGAAGCT 1680
       P  W  S  V  P  H  L  L  H  N  A  Q  C  K  V  R  V  W  K  L

1681 GGCTCAAGCGATGGATCATTTAGTGCGTAACGCCGATGGTGATGAGTTACTTTTTATTCA 1740
       A  Q  A  M  D  H  L  V  R  N  A  D  G  D  E  L  L  F  I  H

1741 TCAAGGAAGCGCTGATTTTTATTGCGATTACGGACACTTGAGCGTTTCACAAGGGGATTA 1800
       Q  G  S  A  D  F  Y  C  D  Y  G  H  L  S  V  S  Q  G  D  Y

1801 TGTGTTGATCCCGCGCTCAACCAATTGGCGGCTGGAGCCAAGTGAGCCGATGTTCATTCT 1860
       V  L  I  P  R  S  T  N  W  R  L  E  P  S  E  P  M  F  I  L
```

```
1861 AATGATAGAGAATACGGATGCGGCGTATGCTTTGCCAGAAAAAGGGCTAGTAGGGAACCA 1920
       M  I  E  N  T  D  A  A  Y  A  L  P  E  K  G  L  V  G  N  H

1921 CGCCATATTCGATCCTGCGGTTTTGCAAGTCCCGTCGATTAACCCAGAGTTTAAAGCGCA 1980
       A  I  F  D  P  A  V  L  Q  V  P  S  I  N  P  E  F  K  A  Q

1981 GTATTCAGAAAAGACCACCCAAGTTCATTTAAAACGTCATGAGAAAATCAGCGTCATTAC 2040
       Y  S  E  K  T  T  Q  V  H  L  K  R  H  E  K  I  S  V  I  T
                                    R2

2041 TTATCCATTTAACCCATTGGATGCAGTAGGTTGGCACGGTGATCTTTCCGTGGTGAAACT 2100
       Y  P  F  N  P  L  D  A  V  G  W  H  G  D  L  S  V  V  K  L

2101 CAACTGGCGAGACATTCGGCCGTTGATGTCGCATCGCTATCATTTGCCGCCCTCCGCTCA 2160
       N  W  R  D  I  R  P  L  M  S  H  R  Y  H  L  P  P  S  A  H

2161 CACAACGTTTGTTGGCCAAGGTTTTGTAGTCTGCACCTTTGTGCCTCGGCCGATTGAGAG 2220
       T  T  F  V  G  Q  G  F  V  V  C  T  F  V  P  R  P  I  E  S

2221 TGACCCCGGCGCTTTAAAGGTTCCGTTTTATCATAACAATGATGATTACGATGAAGTGCT 2280
       D  P  G  A  L  K  V  P  F  Y  H  N  N  D  D  Y  D  E  V  L

2281 CTTCTACCATGCGGGTGATTTCTTTAGCCGCGATAATATTGAAGCTGGCATGGTCACTTT 2340
       F  Y  H  A  G  D  F  F  S  R  D  N  I  E  A  G  M  V  T  F

2341 TCATCCTGCCGGGTTTACCCATGGCCCACATCCGAAAGCGTTCCAAGCTGGGCAAGAGCA 2400
       H  P  A  G  F  T  H  G  P  H  P  K  A  F  Q  A  G  Q  E  H

2401 TAAGAAAAAATTTACCGATGAAGTGGCAGTGATGATTGATACGCGGCATGCATTACACTT 2460
       K  K  K  F  T  D  E  V  A  V  M  I  D  T  R  H  A  L  H  F

2461 TAGTGAGGCGGCGCAGCAGGTTGAAAATCAACAGTACGTCTACAGTTGGAAGAGCGAATA 2520
       S  E  A  A  Q  Q  V  E  N  Q  Q  Y  V  Y  S  W  K  S  E  *
                                                 R

2521 AACGAATAAGAATAAGGAAGTGACATGAAGTTAGCTACGCTAAAAAACGGCACAAGAGAT 2580

2581 GGGTTA 2586
```

Primers:

F AGGCAGTGTCGGTTTTTAATG

R TAACCCATCTCTTGTGCCGT

R2 CCACGGAAAGATCACCGTGCC

Forward hgdA T7: ATCGATGCGAAATTAATACGACTCACTATAGGGGAGATCATTCAACGCAAAGGCAATC

Reverse hgdA T7: AAGCTTAACCCATCTCTTGTGCCGTTTTTTAG

531Ad

1 AGGCAGTGTCGGTTTTTAATGAATGTAGCTAAAATGTTACGCCGTGCTATTTATCTTACT 60

61 GAGCAACTGGTTATGATTGACTTGTGATTAACGTGGCCAATTAAGAAAAAGTTATTAATA 120

121 TTCAGTCAAATAAAAACAAATCATCTCTGTGAAATTCGCTAATTTACTATCCTACTGCTC 180

181 ATTAATAGATTTGTGTAACATTTTAATTACATTGACACTATTTGTTCTCCGCTAAACCGT 240

241 TTTTCGTTATAGGGTTCATTTTTGCTGAAAATTACGCCATGTTCGCAATACTCAATACAA 300

301 GCGTTAACAAAAAAGGAGCGTGAGATGACTTCAACATTTAACCCATTGGGAACAGACGGT 360

361 TTTGAATTTGTGGAGTACACCGCGGCTGATCCCGCTGGAATCGCGCAGTTAAAAGAGCTG 420

421 TTTACTTCTCTTGGCTTTGCTGAGATAGCCAAACATCGCTCAAAAGAGGCGTGGTTATAT 480

481 CGTCAAGGGGATATCAATTTTATCGTTAACTCTCAACCCCATAGCCAAGCTGCAGCGTTT 540

541 GCCAAAGTGCATGGACCTTCAGTGTGTGGGATGGCGTTTCGCGTTCATGATGCATCTGTC 600

601 GCCATGCAGCATGTGCAGGCAAATGGCGGGCGAGAATACAAAACACAAATTGGCCCAATG 660

661 GAACTGAGCATTCCTGCGATTTATGGAATTGGCGATAGCTTGCTTTATTTTGTCGATCGT 720

721 TATGGTAGCCAGAGCATTTATGATGTGGATTTCAAGTTCTATGCCGATGCTAATCAACGA 780

781 ATGACCGACGCCAATGTTGGCTTGTTCGAAATTGACCACTTAACGCATAACGTCAAACAA 840

841 GGCCACATGGACATTTGGTCTGGCTTTTATGAGCGGATTGGTAACTTCCGTGAAATTCGC 900

901 TATTTTGATATTGAAGGGAAGCTAACTGGGTTAGTTAGCCGCGCAATGACAGCGCCTTGC 960

961 GGCAAAATTCGCATCCCAATCAATGAATCGTCTGACGATAAATCACAAATAGAAGAGTTT 1020

1021 ATTCGTGAATACAATGGTGAGGGGATCCAACACATCGCTTTATCCACTGATGATATCTAC 1080

1081 CAGACGGTACGTACTCTGCGCGAACGAGGGATGGATTTCATGCCAACACCGGATACTTAC 1140

1141 TACGAAAAAGTCAGTGATCGAGTTGAAGGGCATCAAGAAGATGTTGATCAGTTAAAAGCG 1200

1201 CTGCGGATCCTGATTGATGGTGCACCGATGAAAGATGGAATTTTGCTGCAAATATTTACT 1260

1261 CAAACCGTCATTGGCCCAGTCTTTTTTGAGATCATTCAACGCAAAGGCAATGAAGGGTTC 1320

1321 GGGGAAGGTAATTTCAAAGCGTTATTTGAGTCGATTGAAGAAGATCAAATTCGACGAGGA 1380

1381 GTACTCAGCGATGCATAAATGGGTCACATTTCCTCATCGGGAAGGGGTATGTTCAAACCA 1440
M H K W V T F P H R E G V C S N Q

1441 AGCTCATGCCGACTTCCCGCCAGAGGCCATCTATGAAAGAGAAGCAGGACGCAGTGGCTT 1500
A H A D F P P E A I Y E R E A G R S G F

1501 TTTTGGCCCTGCGGCCCATTTCCATCACCAACATGCACCGACAGGGTGGAGTGAATGGCA 1560
F G P A A H F H H Q H A P T G W S E W Q

1561 AGGTGAGTTAAGGCCGAGAGCCTTTAATTTTAATTTGATCGAGCAAGCCAAACAAAGTTC 1620
G E L R P R A F N F N L I E Q A K Q S S

1621 ACCGTGGAGCGTGCCGCACCTATTGCATAATGCACAATGTAAAGTACGAGTATGGAAGCT 1680
P W S V P H L L H N A Q C K V R V W K L

1681 GGCTCAAGCGATGGATCATTTAGTGCGTAACGCCGATGGTGATGAGTTACTTTTTATTCA 1740
A Q A M D H L V R N A D G D E L L F I H

1741 TCAAGGAAGCGCTGATTTTTATTGCGATTACGGACACTTGAGCGTTTCACAAGGGGATTA 1800
Q G S A D F Y C D Y G H L S V S Q G D Y

1801 TGTGTTGATCCCGCGCTCAACCAATTGGCGGCTGGAGCCAAGTGAGCCGATGTTCATTCT 1860
V L I P R S T N W R L E P S E P M F I L

1861 AATTTCCCGCCAGAGGCCATCTATGAAAGAGAAGCAGGACGCAGTGGCTTTTTTGGCCCT 1920
I S R Q R P S M K E K Q D A V A F L A L

1921 GCGGCCCATTTCCATCACCAACATGCACCGACAGGGTGGAGTGAATGGCAAGGTGAGTTA 1980
R P I S I T N M H R Q G G V N G K V S *

1981 AGGCCGAGAGCCTTTAATTTTAATTTGATCGAGCAAGCCAAACAAAGTTCACCGTGGAGC 2040

2041 GTGCCGCACCTATTGCATAATGCACAATGTAAAGTACGAGTATGGAAGCTGGCTCAAGCG 2100

2101 ATGGATCATTTAGTGCGTAACGCCGATGGTGATGAGTTACTTTTTATTCATCAAGGAAGC 2160

2161 GCTGATTTTTATTGCGATTACGGACACTTGAGCGTTTCACAAGGGGATTATGTGTTGATC 2220

2221 CCGCGCTCAACCAATTGGCGGCTGGAGCCAAGTGAGCCGATGTTCATTCTAATGATAGAG 2280

2281 AATACGGATGCGGCGTATGCTTTGCCAGAAAAAGGGCTAGTAGGGAACCACGCCATATTC 2340

2341 GATCCTGCGGTTTTGCAAGTCCCGTCGATTAACCCAGAGTTTAAAGCGCAGTATTCAGAA 2400

2401 AAGACCACCCAAGTTCATTTAAAACGTCATGAGAAAATCAGCGTCATTACTTATCCATTT 2460

2461 AACCCATTGGATGCAGTAGGTTGGCACGGTGATCTTTCCGTGGTGAAACTCAACTGGCGA 2520

2521 GACATTCGGCCGTTGATGTCGCATCGCTATCATTTGCCGCCCTCCGCTCACACAACGTTT 2580

2581 GTTGGCCAAGGTTTTGTAGTCTGCACCTTTGTGCCTCGGCCGATTGAGAGTGACCCCGGC 2640

2641 GCTTTAAAGGTTCCGTTTTATCATAACAATGATGATTACGATGAAGTGCTCTTCTACCAT 2700

```
2701 GCGGGTGATTTCTTTAGCCGCGATAATATTGAAGCTGGCATGGTCACTTTTCATCCTGCC 2760

2761 GGGTTTACCCATGGCCCACATCCGAAAGCGTTCCAAGCTGGGCAAGAGCATAAGAAAAAA 2820

2821 TTTACCGATGAAGTGGCAGTGATGATTGATACGCGGCATGCATTACACTTTAGTGAGGCG 2880

2881 GCGCAGCAGGTTGAAAATCAACAGTACGTCTACAGTTGGAAGAGCGAATAAACGAATAAG 2940

2941 AATAAGGAAGTGACATGAAGTTAGCTACGCTAAAAAACGGCACAAGAGATGGGTTA 2996
```

Supplementary Figure S2. Nucleotide sequence of the recombinant clones pP531Ac, pP531Ad, and pP531AcΔhmgA. The figure shows the nucleotide sequences of the region including the genes *hppD* and *hmgA* (531Ac) or *hmgA** (531Ad). The regions highlighted in gray in strain 531Ac and in gray and blue in strain 531Ad indicate the segment that is found in a tandem repeat in strain 531Ad. Yellow highlighted sequences labeled F and R show the sequence of primers used to generate the DNA fragments cloned in the recombinant plasmids pP531Ac and pP531Ad, which were obtained using as template chromosomal DNA from strains 531Ac and 531Ad, respectively. The primer sequence highlighted in green shows the reverse complementary sequence to that of the primer used to generate the recombinant plasmid pP531AcΔhmgA. The sequences of the primers are also shown below the strain 531Ac nucleotide sequence. The figure also shows the sequences of the primers used to generate the plasmid phmgAT7. Nucleotides in blue and green are restriction endonuclease sites, and nucleotides in red are the T7 promoter. Amino acids in red correspond to the 4-hydroxyphenylpyruvate dioxygenase sequence and those in blue correspond to the homogentisate 1,2-dioxygenase sequence. The amino acid sequence of the 4-hydroxyphenylpyruvate dioxygenase were omitted in the 531Ad sequence.

Original pictures Figure 2

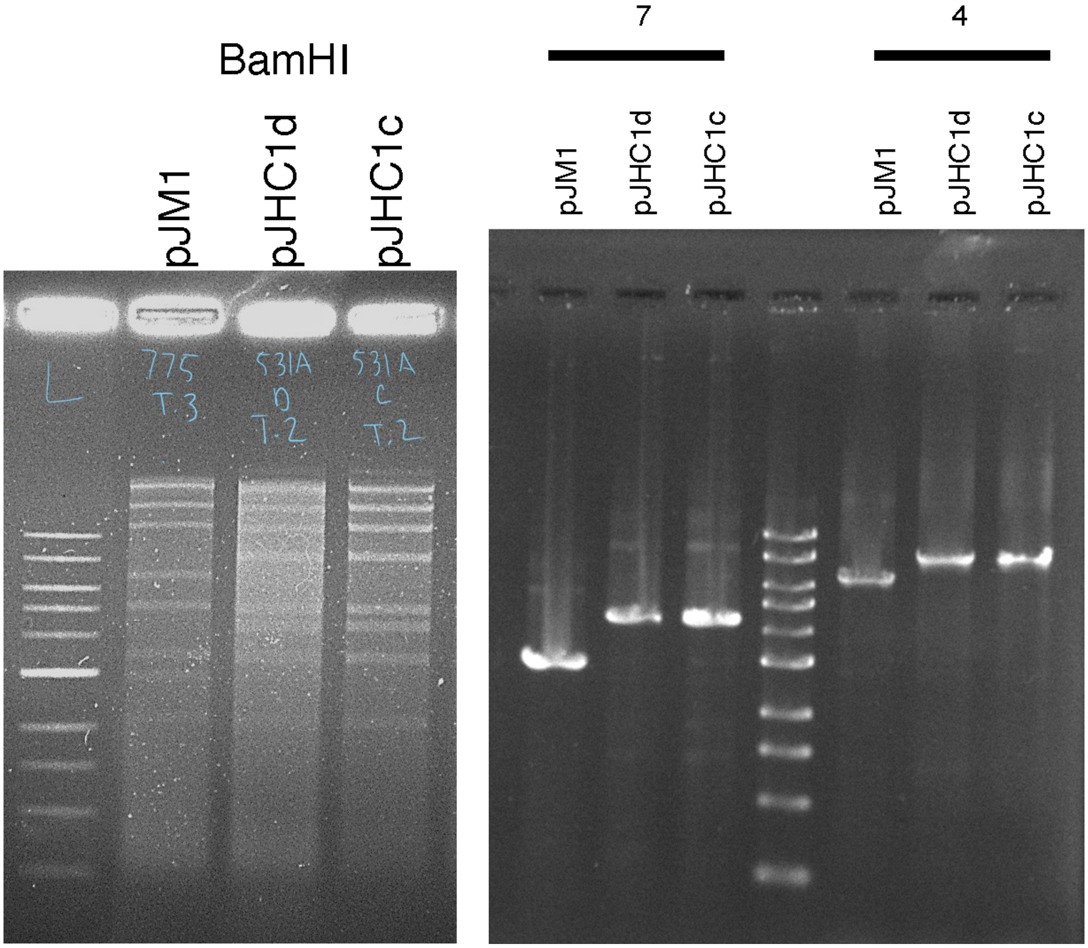

Supplementary Table S1

Pairwise Distance of core-genome phylogeny.

Number of sequence: 75

strain	030305-1_5	12B09	178_90	261_91	425	4299-E1-R1	4299	51_82_2	**531Ac**	**531Ad**	6018_1	601_90	775	87-9-116	87-9-117	90-11-286	90-11-287	9014_8	91-8-178	96F	A023	ATCC-68554	ATCC_14181
030305-1_5	0	0.30567923	0.28849643	0.28847982	0.28883474	0.07655618	0.0765389	0.28855627	0.28834653	0.28865837	0.28951054	0.28850674	0.28947323	0.28843295	0.28838777	0.30726827	0.28855627	0.28852538	0.28842	0.28779653	0.28886201	0.28870416	0.000121
12B09	0.30567923	0	0.25442598	0.25436588	0.25476694	0.30595303	0.30592336	0.25444439	0.25433092	0.25463797	0.25543374	0.25437861	0.25571346	0.25440084	0.25435607	0.31700837	0.25444439	0.25439824	0.25434748	0.25235181	0.25479332	0.25459694	0.30566805
178_90	0.28849643	0.25442598	0	0.00048233	0.00037896	0.28913209	0.28910255	0.00029281	0.00051686	0.00086164	0.00136108	1.72E-05	0.00214667	0.00034455	0.00032718	0.30212641	0.00029286	0.00027575	0.00032721	0.03807833	0.0008783	0.00113697	0.28838343
261_91	0.28847982	0.25436588	0.00048233	0	0.00082702	0.28901015	0.28898061	0.0004652	0.00048252	0.00082737	0.00153393	0.0004652	0.00209524	0.00041356	0.0003962	0.30207867	0.00046524	0.0004482	0.00039623	0.03805304	0.00084414	0.00110278	0.28836679
425	0.28883474	0.25476694	0.00037896	0.00082702	0	0.28938235	0.28935281	0.00063747	0.00086145	0.00120631	0.00170619	0.00036179	0.00249286	0.00068918	0.00067178	0.30246424	0.00063747	0.00062051	0.00067181	0.03842242	0.00122308	0.00148148	0.28871681
4299-E1-R1	0.07655618	0.30595303	0.28913209	0.28901015	0.28938235	0	1.73E-05	0.28916016	0.28887737	0.2891891	0.29009137	0.28912563	0.29026604	0.28903012	0.28902123	0.30988518	0.28908422	0.28905359	0.28905117	0.2869113	0.28946551	0.28923465	0.07660663
4299	0.0765389	0.30592336	0.28910255	0.28898061	0.28935281	1.73E-05	0	0.28913062	0.28884782	0.28915954	0.29006183	0.28909609	0.29023637	0.28900057	0.2889917	0.30985599	0.28905467	0.28902403	0.28902163	0.28688171	0.28943598	0.2892051	0.07658935
51_82_2	0.28855627	0.25444439	0.00029281	0.0004652	0.00063747	0.28916016	0.28913062	0	0.00049973	0.00084458	0.00120604	0.00027564	0.0021298	0.00032739	0.00031003	0.30203378	0.00013781	0.00012064	0.00031005	0.03806897	0.00086125	0.00111998	0.28844325
**531Ac**	0.28834653	0.25433092	0.00051686	0.00048252	0.00086145	0.28887737	0.28884782	0.00049973	0	0.00034466	0.00156861	0.00049971	0.00199127	0.00044805	0.00043072	0.30191151	0.00049974	0.00048272	0.00043073	0.03787866	0.00087872	0.00099936	0.2882235
**531Ad**	0.28865837	0.25463797	0.00086164	0.00082737	0.00120631	0.2891891	0.28915954	0.00084458	0.00034466	0	0.00191383	0.00084454	0.00233815	0.0007929	0.00077547	0.30219127	0.00084459	0.00082771	0.0007755	0.03821546	0.00120638	0.00134429	0.28853536
6018_1	0.28951054	0.25543374	0.00136108	0.00153393	0.00170619	0.29009137	0.29006183	0.00120604	0.00156861	0.00191383	0	0.00134416	0.00316971	0.00139614	0.00137831	0.30297231	0.00120625	0.00118953	0.00137843	0.03906385	0.0019298	0.0021717	0.28939769
601_90	0.28850674	0.25437861	1.72E-05	0.0004652	0.00036179	0.28912563	0.28909609	0.00027564	0.00049971	0.00084454	0.00134416	0	0.00212969	0.00032737	0.00031003	0.30213881	0.00027567	0.00025855	0.00031005	0.038067	0.00086125	0.00111992	0.28839372
775	0.28947323	0.25571346	0.00214667	0.00209524	0.00249286	0.29026604	0.29023637	0.0021298	0.00199127	0.00233815	0.00316971	0.00212969	0	0.00207792	0.00206007	0.30360236	0.0021298	0.0021134	0.00206018	0.03906588	0.00251039	0.00103871	0.28934477
87-9-116	0.28843295	0.25440084	0.00034455	0.00041356	0.00068918	0.28903012	0.28900057	0.00032739	0.00044805	0.0007929	0.00139614	0.00032737	0.00207792	0	5.17E-05	0.30211834	0.00032739	0.0003103	0.00022397	0.03801855	0.00080974	0.00106832	0.28831492
87-9-117	0.28838777	0.25435607	0.00032718	0.0003962	0.00067178	0.28902123	0.2889917	0.00031003	0.00043072	0.00077547	0.00137831	0.00031003	0.00206007	5.17E-05	0	0.30203505	0.00031008	0.00029298	0.00020666	0.03799148	0.00079219	0.00105082	0.28827474
90-11-286	0.30726827	0.31700837	0.30212641	0.30207867	0.30246424	0.30988518	0.30985599	0.30203378	0.30191151	0.30219127	0.30297231	0.30213881	0.30360236	0.30211834	0.30203505	0	0.30203378	0.30204328	0.30205066	0.30104134	0.30247467	0.30230755	0.30727483
90-11-287	0.28855627	0.25444439	0.00029286	0.00046524	0.00063747	0.28908422	0.28905467	0.00013781	0.00049974	0.00084459	0.00120625	0.00027567	0.0021298	0.00032739	0.00031008	0.30203378	0	5.17E-05	0.0003101	0.03806897	0.0008614	0.00111998	0.28844325
9014_8	0.28852538	0.25439824	0.00027575	0.0004482	0.00062051	0.28905359	0.28902403	0.00012064	0.00048272	0.00082771	0.00118953	0.00025855	0.0021134	0.0003103	0.00029298	0.30204328	5.17E-05	0	0.000293	0.03806813	0.00084454	0.00110322	0.28841231
91-8-178	0.28842	0.25434748	0.00032721	0.00039623	0.00067181	0.28905117	0.28902163	0.00031005	0.00043073	0.0007755	0.00137843	0.00031005	0.00206018	0.00022397	0.00020666	0.30205066	0.0003101	0.000293	0	0.03799345	0.00079224	0.00105087	0.28830697
96F	0.28779653	0.25235181	0.03807833	0.03805304	0.03842242	0.2869113	0.28688171	0.03806897	0.03787866	0.03821546	0.03906385	0.038067	0.03906588	0.03801855	0.03799148	0.30104134	0.03806897	0.03806813	0.03799345	0	0.0384085	0.0382865	0.28766839
A023	0.28886201	0.25479332	0.0008783	0.00084414	0.00122308	0.28946551	0.28943598	0.00086125	0.00087872	0.00120638	0.0019298	0.00086125	0.00251039	0.00080974	0.00079219	0.30247467	0.0008614	0.00084454	0.00079224	0.0384085	0	0.00149884	0.28874907
ATCC-68554	0.28870416	0.25459694	0.00113697	0.00110278	0.00148148	0.28923465	0.2892051	0.00111998	0.00099936	0.00134429	0.0021717	0.00111992	0.00103871	0.00106832	0.00105082	0.30230755	0.00111998	0.00110322	0.00105087	0.0382865	0.00149884	0	0.2885862
ATCC_14181	0.000121	0.30566805	0.28838343	0.28836679	0.28871681	0.07660663	0.07658935	0.28844325	0.2882235	0.28853536	0.28939769	0.28839372	0.28934477	0.28831492	0.28827474	0.30727483	0.28844325	0.28841231	0.28830697	0.28766839	0.28874907	0.2885862	0
Ba35-E2-2	0.28826134	0.25412703	0.00062051	0.00058618	0.00096533	0.28879981	0.28877024	0.0006034	0.00048276	0.00082779	0.00167279	0.00060337	0.00180177	0.00055171	0.00053433	0.30188484	0.00060341	0.00058642	0.00053434	0.03781331	0.00098254	0.00079302	0.2881482
Ba35-E2-R1	0.28826928	0.25412789	0.00062052	0.00058625	0.00096553	0.28887391	0.28884435	0.00060341	0.00048286	0.00082796	0.00167282	0.00060341	0.00180215	0.0005518	0.00053434	0.30184383	0.00060351	0.00058652	0.00053437	0.03773487	0.00098256	0.00079319	0.28815612
Ba35-E2-R3	0.28826134	0.25412703	0.00062039	0.00058613	0.00096533	0.28886585	0.28883629	0.00060328	0.00048276	0.00082779	0.00167247	0.00060328	0.00180177	0.00055169	0.00053422	0.30188484	0.00060339	0.0005864	0.00053426	0.03781331	0.00098235	0.00079302	0.2881482
Ba35-E2-R4	0.28826134	0.25412703	0.00062039	0.00058613	0.00096533	0.28886585	0.28883629	0.00060328	0.00048276	0.00082779	0.00167247	0.00060328	0.00180177	0.00055169	0.00053422	0.30188484	0.00060339	0.0005864	0.00053426	0.03781331	0.00098235	0.00079302	0.2881482
Ba35	0.28823753	0.25417745	0.00062013	0.00058582	0.00096474	0.28877569	0.28874614	0.00060303	0.00048247	0.00082729	0.00167178	0.000603	0.00180065	0.00055137	0.000534	0.30186988	0.00060303	0.00058606	0.00053402	0.03780709	0.00098195	0.00079253	0.28812446
CNEVA_NB1	0.29380418	0.27656335	0.24533738	0.24534488	0.24564764	0.29302446	0.29299495	0.24537093	0.24514626	0.24548169	0.2464311	0.2452892	0.24660551	0.24526283	0.24524689	0.3030073	0.24537093	0.24535598	0.24521628	0.24399254	0.24563038	0.24553078	0.29375119
DSM_21597	0.27415392	0.30183263	0.28323959	0.28320447	0.2835893	0.2771228	0.27709302	0.28321196	0.28311298	0.28344037	0.2841631	0.28324647	0.28458203	0.28320677	0.28314843	0.27977844	0.28321425	0.28321564	0.28314583	0.28287781	0.28358698	0.28344119	0.27421085
FF-93	0.29140055	0.25115674	0.22522444	0.22524451	0.22556286	0.2903597	0.29032989	0.22523667	0.22511877	0.22552177	0.22629948	0.22522491	0.22668136	0.22510529	0.22513353	0.3046716	0.22524059	0.22523417	0.22514528	0.22683912	0.22551851	0.22543544	0.29138888
FS-144	0.30624791	0.00173316	0.2552845	0.25522448	0.25563067	0.30682018	0.30679046	0.25530312	0.25518945	0.25549735	0.25629479	0.25523716	0.25646893	0.25525952	0.25521444	0.31806638	0.2553076	0.25526173	0.25520587	0.25318895	0.25565266	0.25540784	0.30623825
HI610	0.00062204	0.30620542	0.28896642	0.28888121	0.28924135	0.07713432	0.07711705	0.28903132	0.28874834	0.2890602	0.2899803	0.28897949	0.28988175	0.28883206	0.2888578	0.30772287	0.28896031	0.28892961	0.28890501	0.28820357	0.28933686	0.28911083	0.00067402
HI618	0.07689779	0.30613712	0.28937906	0.28934537	0.28969523	0.00038025	0.00036297	0.28942181	0.28918475	0.2894969	0.2903594	0.28938951	0.29057141	0.28930992	0.28928763	0.31017494	0.28942181	0.28939129	0.28930263	0.2871943	0.28972754	0.28954739	0.07694038
J360	0.00032836	0.3056281	0.28836285	0.28834621	0.28872108	0.07662001	0.07660273	0.28842265	0.28822289	0.28853468	0.28937678	0.28837314	0.28934892	0.28829934	0.28825918	0.30724037	0.28842764	0.2883967	0.28829638	0.28768284	0.28872838	0.28859049	0.00031113
JLL237	0.29722443	0.31833438	0.3042654	0.30421817	0.30459136	0.29969952	0.29967016	0.30422492	0.30410274	0.30438306	0.30521597	0.30427813	0.30486897	0.30424065	0.30417399	0.21423033	0.30423016	0.30424061	0.30418972	0.30421437	0.30461385	0.30366438	0.29729823
LMG12010	0.28895037	0.25482686	0.00093051	0.00089637	0.00127553	0.28954914	0.2895196	0.00091348	0.00093097	0.00127608	0.00198266	0.00091348	0.00256388	0.00086195	0.00084435	0.302571	0.00091364	0.00089678	0.00084442	0.03851793	0.00039636	0.00155146	0.28883739
M3	0.2888439	0.2548872	0.00041396	0.00086263	0.00068993	0.28944411	0.28941453	0.00067283	0.00089711	0.00124245	0.001743	0.00039678	0.00152426	0.00072461	0.00070718	0.30254348	0.00067283	0.00065586	0.00070721	0.03850867	0.00125923	0.00087967	0.28872581
M93	0.00041474	0.30603267	0.2887257	0.28870915	0.28906398	0.07692972	0.07691244	0.28878558	0.28857089	0.28888274	0.28973982	0.28873605	0.2896935	0.2886573	0.28861704	0.30763383	0.28879057	0.28875979	0.28864928	0.28802102	0.28909125	0.28893343	0.00046664
MHK3	0.29669836	0.28372117	0.26781098	0.26777219	0.26815228	0.29665262	0.2966232	0.2678319	0.26764691	0.26797014	0.26877716	0.26776634	0.2690489	0.26770323	0.26774205	0.29987935	0.26783652	0.26790026	0.26775128	0.26629535	0.26813967	0.2679858	0.2966235
MVAV6203	0.28861845	0.25450191	0.00013785	0.00058599	0.00037908	0.28916641	0.28913686	0.00039639	0.00062043	0.00096535	0.00146539	0.00012064	0.00225124	0.00044811	0.00043077	0.30222015	0.00039639	0.00037932	0.00043079	0.03819097	0.00098223	0.00124067	0.28849046
NB10	0.28857133	0.25458872	0.00041387	0.00048297	0.00075876	0.2892062	0.28917662	0.00039671	0.00051746	0.00086263	0.00146655	0.00039669	0.00214908	0.00031041	0.00029315	0.30225818	0.00039671	0.00037962	0.00029317	0.03811973	0.00087954	0.00113821	0.28845821
NCTC12159	0.27447416	0.30190893	0.28335664	0.28332153	0.28370166	0.27744915	0.27741935	0.28332902	0.28322512	0.2835527	0.28428071	0.28336355	0.28470294	0.28331894	0.28326543	0.28009599	0.28333132	0.28333276	0.28327262	0.28298497	0.28370423	0.28355348	0.27452949
NN_sinnomb	0.28847915	0.2544085	1.72E-05	0.00046511	0.00036173	0.28909527	0.28906574	0.00027558	0.00049963	0.00084441	0.00134385	0	0.00212935	0.00032732	0.00030996	0.30210918	0.00027563	0.00025851	0.00030999	0.03806109	0.00086107	0.00111974	0.28836614
PF4-E1-1	0.28957783	0.25545787	0.22318876	0.2231432	0.22345907	0.28870055	0.28867101	0.22316413	0.22294992	0.22326231	0.22409128	0.22316413	0.22470256	0.22308965	0.22309693	0.29924693	0.22315089	0.22317811	0.22306447	0.22280266	0.2234607	0.22330599	0.28942547
PF4-E1-2	0.28959511	0.25547535	0.22320598	0.22316043	0.2234763	0.28873008	0.28870055	0.22318136	0.22296715	0.22327955	0.22410851	0.22318136	0.22471988	0.22310688	0.22311416	0.29926416	0.22316813	0.22319535	0.22308169	0.2228199	0.22347793	0.22332322	0.28944275
PF4-E1-3	0.28952282	0.25540361	0.22318921	0.22314363	0.22345964	0.28868205	0.28865249	0.22316457	0.22295025	0.22326279	0.22409215	0.22316457	0.22470372	0.22309005	0.22309734	0.2992483	0.22315132	0.22317856	0.22306486	0.22280293	0.22346128	0.22330649	0.28937039
PF4-E1-4	0.28957282	0.25547088	0.22320214	0.22315659	0.22347245	0.28870784	0.2886783	0.22317752	0.22296331	0.2232757	0.22410465	0.22317752	0.22471599	0.22310304	0.22311031	0.299259	0.22316428	0.2231915	0.22307785	0.22281606	0.22347408	0.22331937	0.28942547
PF4-E1-5	0.28957783	0.25545787	0.22318876	0.2231432	0.22345907	0.28879377	0.28876424	0.22316413	0.22294992	0.22326231	0.22409128	0.22316413	0.22470256	0.22308965	0.22309693	0.29924693	0.22315089	0.22317811	0.22306447	0.22280266	0.2234607	0.22330599	0.28942547
PF4-E2-2	0.28957055	0.25545378	0.22315459	0.22315673	0.22346677	0.28861761	0.28858805	0.22314904	0.22296144	0.22327386	0.22406303	0.2231375	0.22471034	0.22309164	0.22306844	0.29925724	0.22314904	0.22317626	0.22304935	0.22281034	0.22344569	0.22331369	0.28942319
PF4-E2-3	0.28957055	0.25545378	0.22315459	0.22315673	0.22346677	0.28861761	0.28858805	0.22314904	0.22296144	0.22327386	0.22406303	0.2231375	0.22471034	0.22309164	0.22306844	0.29925724	0.22314904	0.22317626	0.22304935	0.22281034	0.22344569	0.22331369	0.28942319
PF4-E2-5	0.28957055	0.25545378	0.22315459	0.22315673	0.22346677	0.28861761	0.28858805	0.22314904	0.22296144	0.22327386	0.22406303	0.2231375	0.22471034	0.22309164	0.22306844	0.29925724	0.22314904	0.22317626	0.22304935	0.22281034	0.22344569	0.22331369	0.28942319
PF4-E2-6	0.28957055	0.25545378	0.22315459	0.22315673	0.22346677	0.28861761	0.28858805	0.22314904	0.22296144	0.22327386	0.22406303	0.2231375	0.22471034	0.22309164	0.22306844	0.29925724	0.22314904	0.22317626	0.22304935	0.22281034	0.22344569	0.22331369	0.28942319
PF4-E2-R1	0.28959784	0.25548019	0.22315844	0.22316058	0.22347447	0.28864484	0.28861529	0.22315289	0.22296529	0.2232777	0.22406689	0.22314135	0.22471813	0.22309548	0.22307228	0.29925032	0.22315673	0.22318396	0.2230532	0.22281803	0.22344954	0.22332138	0.28945048
PF4-E2-R4	0.28957556	0.25545378	0.22315844	0.22316058	0.22346677	0.28862259	0.28859304	0.22315289	0.22296144	0.22327386	0.22406689	0.22314135	0.22471034	0.22309164	0.22307228	0.2992624	0.22315289	0.22318011	0.2230532	0.22281034	0.22344954	0.22331369	0.28942319
PF4	0.28957783	0.25545787	0.22317922	0.2231432	0.22345907	0.28867599	0.28864645	0.22316413	0.22294992	0.22326231	0.22409128	0.22316413	0.22470256	0.22308965	0.22309693	0.29924693	0.22315089	0.22317811	0.22306447	0.22280266	0.2234607	0.22330599	0.28942547
PF430-3	0.28959511	0.25547535	0.22320598	0.22316043	0.2234763	0.28871781	0.28868827	0.22318136	0.22296715	0.22327955	0.22410851	0.22318136	0.22471988	0.22310688	0.22311416	0.29926416	0.22316813	0.22319535	0.22308169	0.2228199	0.22347793	0.22332322	0.28944275
PF4_substr_	0.28965791	0.25547649	0.2232506	0.22325277	0.22358578	0.28868009	0.28865053	0.22324507	0.22307653	0.22338903	0.22415934	0.22323352	0.22483024	0.22318381	0.22316443	0.29931222	0.22324507	0.22327234	0.22317212	0.22292346	0.22354178	0.22343268	0.28950053
PF4_substr_	0.29001176	0.25573868	0.22364041	0.22364267	0.22397585	0.28903293	0.28900335	0.22363495	0.22346629	0.22377912	0.22455002	0.22362338	0.22522319	0.22357366	0.22355417	0.29950149	0.22363495	0.22366241	0.22356188	0.2233189	0.22393183	0.22381495	0.28986661
PF4_substr_	0.2900064	0.25593146	0.22357625	0.2235785	0.22391147	0.28902822	0.28899867	0.22357079	0.22340224	0.22371484	0.22448522	0.22355923	0.22515765	0.22350953	0.22349007	0.29965523	0.22357079	0.2235982	0.22349778	0.22326263	0.22386748	0.22375838	0.28986134
PF7	0.29118933	0.26201919	0.22665886	0.22668528	0.22698372	0.29027581	0.2902463	0.22667126	0.22647525	0.22677125	0.22772397	0.2266368	0.22784657	0.2265975	0.22657273	0.30221939	0.22669309	0.22668735	0.22653666	0.22596717	0.22703625	0.22686541	0.29130157
RV22	0.00103005	0.30659315	0.29085066	0.2908697	0.29122128	0.07848848	0.07847072	0.29085582	0.29073795	0.29105896	0.29181912	0.29086163	0.29186808	0.29082901	0.29079223	0.30827829	0.29087358	0.29084288	0.29081805	0.29007444	0.29124421	0.29111249	0.00115457
S2_2_9	0.29295181	0.31721727	0.30197457	0.30194402	0.30229513	0.29366874	0.29363928	0.30198532	0.30177688	0.30203934	0.30294097	0.30195247	0.30313788	0.30194923	0.30188322	0.2753326	0.30198532	0.30199479	0.30189882	0.30091547	0.30232278	0.30211439	0.29306441
S3_4_9	0.29531396	0.3169845	0.30112178	0.30109102	0.30144233	0.29734116	0.29731176	0.30113235	0.30091866	0.30118093	0.30208782	0.30109955	0.30239163	0.30109621	0.30103043	0.27387558	0.30113235	0.30114146	0.30104599	0.30073101	0.30146995	0.3012615	0.29542391
T265	0.28840434	0.2542364	0.00072389	0.00068958	0.00106863	0.28895262	0.28892306	0.0007068	0.00058615	0.00093116	0.00177617	0.00070676	0.00190549	0.0006551	0.00063771	0.30199155	0.0007068	0.00068986	0.00063773	0.03792949	0.00108591	0.00089635	0.28828626
VA1	0.2897248	0.25567328	0.00172274	0.00168887	0.00206779	0.29032537	0.29029584	0.00170587	0.00172348	0.00206868	0.00277505	0.00170578	0.00335971	0.00165443	0.0016366	0.30325283	0.00170607	0.00168957	0.00163672	0.0392992	0.00208466	0.0023437	0.28961201
VIB12	0.28490923	0.29782571	0.27898212	0.27895756	0.27928489	0.23212584	0.23209521	0.27902272	0.27882404	0.27916832	0.2799917	0.27895638	0.28040452	0.2789278	0.27892544	0.30138502	0.27898732	0.27900419	0.27893991	0.27598734	0.27936448	0.27910154	0.28484964
VIB43	0.00096749	0.30581206	0.28859107	0.28857449	0.28894915	0.07654436	0.07652709	0.28865089	0.28845124	0.28876294	0.28960464	0.28860139	0.28957784	0.28852765	0.28848744	0.30738137	0.28865588	0.28862505	0.28852465	0.28783034	0.28895644	0.28881864	0.00095036
VIB_18	0.28845456	0.25438244	0.00036163	0.00043067	0.00070626	0.28907073	0.28904119	0.00034449	0.00046519	0.00080997	0.00141282	0.00034449	0.0020948	0.00025842	0.00024109	0.30208513	0.00034455	0.00032746	3.44E-05	0.03802793	0.00082666	0.00108533	0.28834154
VIB_93	0.28852538	0.25439824	0.00027575	0.0004482	0.00062051	0.28905359	0.28902403	0.00012064	0.00048272	0.00082771	0.00118953	0.00025855	0.0021134	0.0003103	0.00029298	0.30204328	5.17E-05	0	0.000293	0.03806813	0.00084454	0.00110322	0.28841231

strain	Ba35-E2-2	Ba35-E2-R1	Ba35-E2-R3	Ba35-E2-R4	Ba35	CNEVA_NB1	DSM_21597	FF-93	FS-144	HI610	HI618	J360	JLL237	LMG12010	M3	M93	MHK3	MVAV6203	NB10	NCTC12159	NN_sinnomb	PF4-E1-1	PF4-E1-2	PF4-E1-3	PF4-E1-4	PF4-E1-5
030305-1_5	0.28826134	0.28826928	0.28826134	0.28826134	0.28823753	0.29380418	0.27415392	0.29140055	0.30624791	0.00062204	0.07689779	0.00032836	0.29722443	0.28895037	0.2888439	0.00041474	0.29669836	0.28861845	0.28857133	0.27447416	0.28847915	0.28957783	0.28959511	0.28952282	0.28957282	0.28957783
12B09	0.25412703	0.25412789	0.25412703	0.25412703	0.25417745	0.27656335	0.30183263	0.25115674	0.00173316	0.30620542	0.30613712	0.3056281	0.31833438	0.25482686	0.2548872	0.30603267	0.28372117	0.25450191	0.25458872	0.30190893	0.2544085	0.25545787	0.25547535	0.25540361	0.25547088	0.25545787
178_90	0.00062051	0.00062052	0.00062039	0.00062039	0.00062013	0.24533738	0.28323959	0.22522444	0.2552845	0.28896642	0.28937906	0.28836285	0.3042654	0.00093051	0.00041396	0.2887257	0.26781098	0.00013785	0.00041387	0.28335664	1.72E-05	0.22318876	0.22320598	0.22318921	0.22320214	0.22318876
261_91	0.00058618	0.00058625	0.00058613	0.00058613	0.00058582	0.24534488	0.28320447	0.22524451	0.25522448	0.28888121	0.28934537	0.28834621	0.30421817	0.00089637	0.00086263	0.28870915	0.26777219	0.00058599	0.00048297	0.28332153	0.00046511	0.2231432	0.22316043	0.22314363	0.22315659	0.2231432
425	0.00096533	0.00096553	0.00096533	0.00096533	0.00096474	0.24564764	0.2835893	0.22556286	0.25563067	0.28924135	0.28969523	0.28872108	0.30459136	0.00127553	0.00068993	0.28906398	0.26815228	0.00037908	0.00075876	0.28370166	0.00036173	0.22345907	0.2234763	0.22345964	0.22347245	0.22345907
4299-E1-R1	0.28879981	0.28887391	0.28886585	0.28886585	0.28877569	0.29302446	0.2771228	0.2903597	0.30682018	0.07713432	0.00038025	0.07662001	0.29969952	0.28954914	0.28944411	0.07692972	0.29665262	0.28916641	0.2892062	0.27744915	0.28909527	0.28870055	0.28873008	0.28868205	0.28870784	0.28879377
4299	0.28877024	0.28884435	0.28883629	0.28883629	0.28874614	0.29299495	0.27709302	0.29032989	0.30679046	0.07711705	0.00036297	0.07660273	0.29967016	0.2895196	0.28941453	0.07691244	0.2966232	0.28913686	0.28917662	0.27741935	0.28906574	0.28867101	0.28870055	0.28865249	0.2886783	0.28876424
51_82_2	0.0006034	0.00060341	0.00060328	0.00060328	0.00060303	0.24537093	0.28321196	0.22523667	0.25530312	0.28903132	0.28942181	0.28842265	0.30422492	0.00091348	0.00067283	0.28878558	0.2678319	0.00039639	0.00039671	0.28332902	0.00027558	0.22316413	0.22318136	0.22316457	0.22317752	0.22316413
**531Ac**	0.00048276	0.00048286	0.00048276	0.00048276	0.00048247	0.24514626	0.28311298	0.22511877	0.25518945	0.28874834	0.28918475	0.28822289	0.30410274	0.00093097	0.00089711	0.28857089	0.26764691	0.00062043	0.00051746	0.28322512	0.00049963	0.22294992	0.22296715	0.22295025	0.22296331	0.22294992
**531Ad**	0.00082779	0.00082796	0.00082779	0.00082779	0.00082729	0.24548169	0.28344037	0.22552177	0.25549735	0.2890602	0.2894969	0.28853468	0.30438306	0.00127608	0.00124245	0.28888274	0.26797014	0.00096535	0.00086263	0.2835527	0.00084441	0.22326231	0.22327955	0.22326279	0.2232757	0.22326231
6018_1	0.00167279	0.00167282	0.00167247	0.00167247	0.00167178	0.2464311	0.2841631	0.22629948	0.25629479	0.2899803	0.2903594	0.28937678	0.30521597	0.00198266	0.001743	0.28973982	0.26877716	0.00146539	0.00146655	0.28428071	0.00134385	0.22409128	0.22410851	0.22409215	0.22410465	0.22409128
601_90	0.00060337	0.00060341	0.00060328	0.00060328	0.000603	0.2452892	0.28324647	0.22522491	0.25523716	0.28897949	0.28938951	0.28837314	0.30427813	0.00091348	0.00039678	0.28873605	0.26776634	0.00012064	0.00039669	0.28336355	0	0.22316413	0.22318136	0.22316457	0.22317752	0.22316413
775	0.00180177	0.00180215	0.00180177	0.00180177	0.00180065	0.24660551	0.28458203	0.22668136	0.25646893	0.28988175	0.29057141	0.28934892	0.30486897	0.00256388	0.00152426	0.2896935	0.2690489	0.00225124	0.00214908	0.28470294	0.00212935	0.22470256	0.22471988	0.22470372	0.22471599	0.22470256
87-9-116	0.00055171	0.0005518	0.00055169	0.00055169	0.00055137	0.24526283	0.28320677	0.22510529	0.25525952	0.28883206	0.28930992	0.28829934	0.30424065	0.00086195	0.00072461	0.2886573	0.26770323	0.00044811	0.00031041	0.28331894	0.00032732	0.22308965	0.22310688	0.22309005	0.22310304	0.22308965
87-9-117	0.00053433	0.00053434	0.00053422	0.00053422	0.000534	0.24524689	0.28314843	0.22513353	0.25521444	0.2888578	0.28928763	0.28825918	0.30417399	0.00084435	0.00070718	0.28861704	0.26774205	0.00043077	0.00029315	0.28326543	0.00030996	0.22309693	0.22311416	0.22309734	0.22311031	0.22309693
90-11-286	0.30188484	0.30184383	0.30188484	0.30188484	0.30186988	0.3030073	0.27977844	0.3046716	0.31806638	0.30772287	0.31017494	0.30724037	0.21423033	0.302571	0.30254348	0.30763383	0.29987935	0.30222015	0.30225818	0.28009599	0.30210918	0.29924693	0.29926416	0.2992483	0.299259	0.29924693
90-11-287	0.00060341	0.00060351	0.00060339	0.00060339	0.00060303	0.24537093	0.28321425	0.22524059	0.2553076	0.28896031	0.28942181	0.28842764	0.30423016	0.00091364	0.00067283	0.28879057	0.26783652	0.00039639	0.00039671	0.28333132	0.00027563	0.22315089	0.22316813	0.22315132	0.22316428	0.22315089
9014_8	0.00058642	0.00058652	0.0005864	0.0005864	0.00058606	0.24535598	0.28321564	0.22523417	0.25526173	0.28892961	0.28939129	0.2883967	0.30424061	0.00089678	0.00065586	0.28875979	0.26790026	0.00037932	0.00037962	0.28333276	0.00025851	0.22317811	0.22319535	0.22317856	0.2231915	0.22317811
91-8-178	0.00053434	0.00053437	0.00053426	0.00053426	0.00053402	0.24521628	0.28314583	0.22514528	0.25520587	0.28890501	0.28930263	0.28829638	0.30418972	0.00084442	0.00070721	0.28864928	0.26775128	0.00043079	0.00029317	0.28327262	0.00030999	0.22306447	0.22308169	0.22306486	0.22307785	0.22306447
96F	0.03781331	0.03773487	0.03781331	0.03781331	0.03780709	0.24399254	0.28287781	0.22683912	0.25318895	0.28820357	0.2871943	0.28768284	0.30421437	0.03851793	0.03850867	0.28802102	0.26629535	0.03819097	0.03811973	0.28298497	0.03806109	0.22280266	0.2228199	0.22280293	0.22281606	0.22280266
A023	0.00098254	0.00098256	0.00098235	0.00098235	0.00098195	0.24563038	0.28358698	0.22551851	0.25565266	0.28933686	0.28972754	0.28872838	0.30461385	0.00039636	0.00125923	0.28909125	0.26813967	0.00098223	0.00087954	0.28370423	0.00086107	0.2234607	0.22347793	0.22346128	0.22347408	0.2234607
ATCC-68554	0.00079302	0.00079319	0.00079302	0.00079302	0.00079253	0.24553078	0.28344119	0.22543544	0.25540784	0.28911083	0.28954739	0.28859049	0.30366438	0.00155146	0.00087967	0.28893343	0.2679858	0.00124067	0.00113821	0.28355348	0.00111974	0.22330599	0.22332322	0.22330649	0.22331937	0.22330599
ATCC_14181	0.2881482	0.28815612	0.2881482	0.2881482	0.28812446	0.29375119	0.27421085	0.29138888	0.30623825	0.00067402	0.07694038	0.00031113	0.29729823	0.28883739	0.28872581	0.00046664	0.2966235	0.28849046	0.28845821	0.27452949	0.28836614	0.28942547	0.28944275	0.28937039	0.28942547	0.28942547
Ba35-E2-2	0	0	0	0	0	0.24513088	0.2829713	0.22488813	0.25498546	0.28867067	0.28912724	0.28813764	0.30399089	0.00103484	0.00100116	0.2884908	0.26759567	0.00072424	0.00062127	0.28309808	0.00060327	0.22284295	0.2228602	0.22284324	0.22285635	0.22284295
Ba35-E2-R1	0	0	0	0	0	0.24513867	0.28292894	0.22491771	0.2549865	0.28874481	0.28913536	0.28813559	0.30398482	0.00103486	0.00100136	0.28849879	0.26758207	0.00072439	0.0006214	0.28304593	0.00060328	0.22288128	0.22289853	0.22288159	0.22289468	0.22288128
Ba35-E2-R3	0	0	0	0	0	0.24515695	0.28297391	0.22488813	0.25498546	0.28873678	0.28912724	0.28812768	0.30399089	0.00103464	0.00100116	0.2884908	0.26759567	0.00072424	0.00062127	0.2830909	0.00060316	0.22288689	0.22290413	0.2228872	0.22290029	0.22288689
Ba35-E2-R4	0	0	0	0	0	0.24515695	0.28297391	0.22488813	0.25498546	0.28873678	0.28912724	0.28812768	0.30399089	0.00103464	0.00100116	0.2884908	0.26759567	0.00072424	0.00062127	0.2830909	0.00060316	0.22288689	0.22290413	0.2228872	0.22290029	0.22288689
Ba35	0	0	0	0	0	0.24504763	0.2829557	0.22495998	0.25504002	0.2886516	0.28910289	0.2881189	0.30396263	0.00103422	0.00100053	0.28847184	0.26757213	0.00072379	0.00062088	0.2830824	0.00060291	0.22286364	0.22288087	0.22286394	0.22287703	0.22286364
CNEVA_NB1	0.24513088	0.24513867	0.24515695	0.24515695	0.24504763	0	0.28395275	0.24540925	0.27725846	0.29428655	0.29317387	0.29371464	0.30401173	0.24557163	0.24568496	0.29405048	0.26741798	0.24537453	0.24509431	0.28429746	0.24532013	0.23639626	0.23641351	0.23652369	0.23640943	0.23639626
DSM_21597	0.2829713	0.28292894	0.28297391	0.28297391	0.2829557	0.28395275	0	0.28776051	0.30114184	0.27473839	0.2773518	0.27412239	0.27261903	0.28362124	0.28366049	0.27450607	0.28380827	0.2833716	0.2833031	0.00024174	0.28322233	0.28529772	0.28531497	0.28532712	0.28531005	0.28529772
FF-93	0.22488813	0.22491771	0.22488813	0.22488813	0.22495998	0.24540925	0.28776051	0	0.25137285	0.29197488	0.29050952	0.29134023	0.30568704	0.22569076	0.22569517	0.2916863	0.27301681	0.225295	0.22522209	0.28793584	0.22520704	0.23229915	0.23231655	0.23239093	0.23231251	0.23229915
FS-144	0.25498546	0.2549865	0.25498546	0.25498546	0.25504002	0.27725846	0.30114184	0.25137285	0	0.3067643	0.30700706	0.30619808	0.31868902	0.2556868	0.25569975	0.30659806	0.28367204	0.2553609	0.25545308	0.30123717	0.25526698	0.25637118	0.2563887	0.25631725	0.25638421	0.25637118
HI610	0.28867067	0.28874481	0.28873678	0.28873678	0.2886516	0.29428655	0.27473839	0.29197488	0.3067643	0	0.07748733	0.00088115	0.29790065	0.28942047	0.28925105	0.00096752	0.29715017	0.28902523	0.28897845	0.27504629	0.28894915	0.29012868	0.29015821	0.29008623	0.29013593	0.29018497
HI618	0.28912724	0.28913536	0.28912724	0.28912724	0.28910289	0.29317387	0.2773518	0.29050952	0.30700706	0.07748733	0	0.07696167	0.29995505	0.28981646	0.28975748	0.0772651	0.29677341	0.28946913	0.28947141	0.27767195	0.28936178	0.28879631	0.28881359	0.28874092	0.28879631	0.28879631
J360	0.28813764	0.28813559	0.28812768	0.28812768	0.2881189	0.29371464	0.27412239	0.29134023	0.30619808	0.00088115	0.07696167	0	0.29738184	0.28881665	0.28873008	0.0006739	0.2966087	0.28850475	0.28845755	0.27444091	0.28834557	0.2896342	0.28965148	0.28957923	0.28962919	0.2896342
JLL237	0.30399089	0.30398482	0.30399089	0.30399089	0.30396263	0.30401173	0.27261903	0.30568704	0.31868902	0.29790065	0.29995505	0.29738184	0	0.30472873	0.30401243	0.29765505	0.29774874	0.30434108	0.30441534	0.27301922	0.30424817	0.29916415	0.29918139	0.29914825	0.29917623	0.29916415
LMG12010	0.00103484	0.00103486	0.00103464	0.00103464	0.00103422	0.24557163	0.28362124	0.22569076	0.2556868	0.28942047	0.28981646	0.28881665	0.30472873	0	0.00131177	0.28917975	0.26824978	0.00103455	0.00093184	0.28379686	0.00091327	0.22364338	0.22366062	0.22364405	0.22365677	0.22364338
M3	0.00100116	0.00100136	0.00100116	0.00100116	0.00100053	0.24568496	0.28366049	0.22569517	0.25569975	0.28925105	0.28975748	0.28873008	0.30401243	0.00131177	0	0.28907345	0.26837518	0.0004486	0.00079432	0.28377304	0.00039672	0.22362178	0.22363903	0.22362243	0.22363517	0.22362178
M93	0.2884908	0.28849879	0.2884908	0.2884908	0.28847184	0.29405048	0.27450607	0.2916863	0.30659806	0.00096752	0.0772651	0.0006739	0.29765505	0.28917975	0.28907345	0	0.29702029	0.2888428	0.28880585	0.27482475	0.28870842	0.2898711	0.28988838	0.28981624	0.2898711	0.2898711
MHK3	0.26759567	0.26758207	0.26759567	0.26759567	0.26757213	0.26741798	0.28380827	0.27301681	0.28367204	0.29715017	0.29677341	0.2966087	0.29774874	0.26824978	0.26837518	0.29702029	0	0.26788608	0.26781925	0.28401948	0.26779375	0.26560723	0.26562446	0.26561018	0.26561988	0.26560723
MVAV6203	0.00072424	0.00072439	0.00072424	0.00072424	0.00072379	0.24537453	0.2833716	0.225295	0.2553609	0.28902523	0.28946913	0.28850475	0.30434108	0.00103455	0.0004486	0.2888428	0.26788608	0	0.00051749	0.28347898	0.00012062	0.22318751	0.22320474	0.22318796	0.2232009	0.22318751
NB10	0.00062127	0.0006214	0.00062127	0.00062127	0.00062088	0.24509431	0.2833031	0.22522209	0.25545308	0.28897845	0.28947141	0.28845755	0.30441534	0.00093184	0.00079432	0.28880585	0.26781925	0.00051749	0	0.28342033	0.00039663	0.22314035	0.2231576	0.22314078	0.22315375	0.22314035
NCTC12159	0.28309808	0.28304593	0.2830909	0.2830909	0.2830824	0.28429746	0.00024174	0.28793584	0.30123717	0.27504629	0.27767195	0.27444091	0.27301922	0.28379686	0.28377304	0.27482475	0.28401948	0.28347898	0.28342033	0	0.28333938	0.2856107	0.28562797	0.28564026	0.28562304	0.2856107
NN_sinnomb	0.00060327	0.00060328	0.00060316	0.00060316	0.00060291	0.24532013	0.28322233	0.22520704	0.25526698	0.28894915	0.28936178	0.28834557	0.30424817	0.00091327	0.00039672	0.28870842	0.26779375	0.00012062	0.00039663	0.28333938	0	0.22316584	0.22318306	0.22316628	0.22317922	0.22316584
PF4-E1-1	0.22284295	0.22288128	0.22288689	0.22288689	0.22286364	0.23639626	0.28529772	0.23229915	0.25637118	0.29012868	0.28879631	0.2896342	0.29916415	0.22364338	0.22362178	0.2898711	0.26560723	0.22318751	0.22314035	0.2856107	0.22316584	0	1.72E-05	1.72E-05	0	0
PF4-E1-2	0.2228602	0.22289853	0.22290413	0.22290413	0.22288087	0.23641351	0.28531497	0.23231655	0.2563887	0.29015821	0.28881359	0.28965148	0.29918139	0.22366062	0.22363903	0.28988838	0.26562446	0.22320474	0.2231576	0.28562797	0.22318306	1.72E-05	0	3.45E-05	1.72E-05	1.72E-05
PF4-E1-3	0.22284324	0.22288159	0.2228872	0.2228872	0.22286394	0.23652369	0.28532712	0.23239093	0.25631725	0.29008623	0.28874092	0.28957923	0.29914825	0.22364405	0.22362243	0.28981624	0.26561018	0.22318796	0.22314078	0.28564026	0.22316628	1.72E-05	3.45E-05	0	1.72E-05	1.72E-05
PF4-E1-4	0.22285635	0.22289468	0.22290029	0.22290029	0.22287703	0.23640943	0.28531005	0.23231251	0.25638421	0.29013593	0.28879631	0.28962919	0.29917623	0.22365677	0.22363517	0.2898711	0.26561988	0.2232009	0.22315375	0.28562304	0.22317922	0	1.72E-05	1.72E-05	0	0
PF4-E1-5	0.22284295	0.22288128	0.22288689	0.22288689	0.22286364	0.23639626	0.28529772	0.23229915	0.25637118	0.29018497	0.28879631	0.2896342	0.29916415	0.22364338	0.22362178	0.2898711	0.26560723	0.22318751	0.22314035	0.2856107	0.22316584	0	1.72E-05	1.72E-05	0	0
PF4-E2-2	0.22284492	0.22286611	0.22287173	0.22287173	0.22286177	0.23638716	0.28530752	0.23231128	0.25637163	0.29007066	0.288794	0.28963193	0.29921409	0.22361501	0.22362949	0.28987386	0.26562096	0.2231952	0.22314805	0.28562303	0.22313736	1.72E-05	3.45E-05	3.45E-05	1.72E-05	1.72E-05
PF4-E2-3	0.22284492	0.22286611	0.22287173	0.22287173	0.22286177	0.23638716	0.28530752	0.23231128	0.25637163	0.29007066	0.288794	0.28963193	0.29921409	0.22361501	0.22362949	0.28987386	0.26562096	0.2231952	0.22314805	0.28562303	0.22313736	1.72E-05	3.45E-05	3.45E-05	1.72E-05	1.72E-05
PF4-E2-5	0.22284492	0.22286611	0.22287173	0.22287173	0.22286177	0.23638716	0.28530752	0.23231128	0.25637163	0.29007066	0.288794	0.28963193	0.29921409	0.22361501	0.22362949	0.28987386	0.26562096	0.2231952	0.22314805	0.28562303	0.22313736	1.72E-05	3.45E-05	3.45E-05	1.72E-05	1.72E-05
PF4-E2-6	0.22284492	0.22286611	0.22287173	0.22287173	0.22286177	0.23638716	0.28530752	0.23231128	0.25637163	0.29007066	0.288794	0.28963193	0.29921409	0.22361501	0.22362949	0.28987386	0.26562096	0.2231952	0.22314805	0.28562303	0.22313736	1.72E-05	3.45E-05	3.45E-05	1.72E-05	1.72E-05
PF4-E2-R1	0.22284877	0.22286996	0.22287557	0.22287557	0.22286945	0.23639532	0.28531244	0.23231532	0.25639364	0.29009295	0.28882127	0.28965422	0.29921925	0.22361887	0.22363721	0.28989614	0.26564278	0.2232029	0.22315575	0.28562796	0.22314121	3.45E-05	5.17E-05	5.17E-05	3.45E-05	3.45E-05
PF4-E2-R4	0.22284877	0.22286996	0.22287557	0.22287557	0.22286561	0.23639124	0.28531244	0.23231128	0.25637163	0.29007568	0.288794	0.28963694	0.29920201	0.22361887	0.22362949	0.28987386	0.26562096	0.2231952	0.2231519	0.28562303	0.22314121	1.72E-05	3.45E-05	3.45E-05	1.72E-05	1.72E-05
PF4	0.22284295	0.22288128	0.22288689	0.22288689	0.22286364	0.23639626	0.28529772	0.23229915	0.25637118	0.29012868	0.28879631	0.2896342	0.29916415	0.22364338	0.22362178	0.2898711	0.26560723	0.22318751	0.22314035	0.2856107	0.22316584	0	1.72E-05	1.72E-05	0	0
PF430-3	0.2228602	0.22289853	0.22290413	0.22290413	0.22288087	0.23641351	0.28531497	0.23231655	0.2563887	0.29014595	0.28881359	0.28965148	0.29918139	0.22366062	0.22363903	0.28988838	0.26562446	0.22320474	0.2231576	0.28562797	0.22318306	1.72E-05	0	3.45E-05	1.72E-05	1.72E-05
PF4_substr_	0.22296773	0.22296213	0.22296773	0.22296773	0.2229845	0.23648287	0.28544551	0.23239461	0.25639453	0.29013359	0.28887121	0.28969475	0.29932078	0.22371122	0.22374871	0.28995627	0.26569315	0.22331034	0.22327098	0.28573156	0.22323337	0.0001034	0.00012063	0.00012069	0.00010339	0.0001034
PF4_substr_	0.22335761	0.22335209	0.22335761	0.22335761	0.22337416	0.23682711	0.28583027	0.23273475	0.25667099	0.29048772	0.28923689	0.29004861	0.29983094	0.22410163	0.22413942	0.29032258	0.26590293	0.223706	0.22365354	0.28612907	0.22362316	0.00048288	0.00050012	0.00050035	0.00048287	0.00048288
PF4_substr_	0.2232936	0.22328807	0.2232936	0.2232936	0.22331018	0.23690932	0.28572415	0.23279373	0.25686354	0.29048203	0.28923205	0.29004322	0.29968106	0.22403712	0.22407484	0.290317	0.26603448	0.2236495	0.22359702	0.28602269	0.22355902	0.00049978	0.00051702	0.00051726	0.00049978	0.00049978
PF7	0.22635263	0.22633929	0.22636143	0.22636143	0.22638843	0.18759273	0.2856921	0.23507203	0.26294681	0.2919437	0.29030976	0.29134919	0.30572645	0.22700084	0.22694411	0.29159108	0.27085955	0.22673056	0.22661722	0.28603981	0.22664163	0.22589147	0.2259087	0.22587595	0.22590481	0.22590647
RV22	0.29059541	0.29059373	0.29058508	0.29058508	0.29056946	0.29495176	0.27525693	0.29267813	0.3070991	0.00166895	0.07882386	0.00134962	0.2966186	0.29133648	0.291234	0.00131371	0.29757908	0.2909895	0.29093365	0.27557192	0.2908329	0.29161932	0.29163707	0.29156378	0.29161932	0.29161932
S2_2_9	0.30178464	0.30177811	0.30178464	0.30178464	0.30166635	0.29949098	0.25037098	0.30574745	0.31808867	0.29356483	0.29388785	0.29307991	0.21554636	0.30245349	0.30221885	0.29334117	0.29527552	0.3020494	0.30211658	0.25066036	0.30195734	0.29809233	0.29810957	0.29816213	0.29810443	0.29809233
S3_4_9	0.300776	0.30076926	0.300776	0.300776	0.30077896	0.30233963	0.25223018	0.30857968	0.31794378	0.29592648	0.29757851	0.29544905	0.21012719	0.30151395	0.30138215	0.29570506	0.29532527	0.30118592	0.30126281	0.25252473	0.30110454	0.2992107	0.29922793	0.29926378	0.2992278	0.2992107
T265	0.00017248	0.00017252	0.00017248	0.00017248	0.00017238	0.2452436	0.28322542	0.22517801	0.25509954	0.28881129	0.2892652	0.28828564	0.30387846	0.0011383	0.00110459	0.28863377	0.26770594	0.0008276	0.00072474	0.28333765	0.00070665	0.22309005	0.22310729	0.22309046	0.22310345	0.22309005
VA1	0.00182771	0.00182784	0.00182746	0.00182746	0.00182661	0.2464446	0.28432354	0.22640327	0.25651717	0.29019716	0.2905909	0.28959104	0.30535825	0.00213764	0.00210508	0.28995402	0.26889061	0.00182715	0.00172509	0.28444122	0.00170551	0.22433047	0.22434771	0.22433145	0.22434384	0.22433047
VIB12	0.27863038	0.2786247	0.27865336	0.27865336	0.27868654	0.28013227	0.28102708	0.28183326	0.29849776	0.28549142	0.23218219	0.28479784	0.3006502	0.27944713	0.27929846	0.28522885	0.27752413	0.27902593	0.27903072	0.2812749	0.27896484	0.27968329	0.27970057	0.27962364	0.27969574	0.27968329
VIB43	0.28836614	0.28836413	0.28835618	0.28835618	0.28834727	0.29396403	0.27441756	0.29149296	0.30637685	0.00151994	0.07688449	0.00098463	0.29750881	0.28904481	0.28895846	0.00131281	0.29673596	0.28872801	0.288686	0.27473136	0.2885738	0.28959596	0.28961324	0.28954099	0.28959096	0.28959596
VIB_18	0.00056882	0.00056883	0.00056871	0.00056871	0.00056848	0.24527683	0.28318034	0.22518008	0.2552409	0.28892459	0.28933721	0.28832098	0.30422419	0.00087884	0.00074171	0.28868384	0.26778574	0.00046525	0.00032766	0.28329737	0.00034441	0.22313907	0.2231563	0.2231395	0.22315245	0.22313907
VIB_93	0.00058642	0.00058652	0.0005864	0.0005864	0.00058606	0.24535598	0.28321564	0.22523417	0.25526173	0.28892961	0.28939129	0.2883967	0.30424061	0.00089678	0.00065586	0.28875979	0.26790026	0.00037932	0.00037962	0.28333276	0.00025851	0.22317811	0.22319535	0.22317856	0.2231915	0.22317811

strain	PF4-E2-2	PF4-E2-3	PF4-E2-5	PF4-E2-6	PF4-E2-R1	PF4-E2-R4	PF4	PF430-3	PF4_substr_	PF4_substr_	PF4_substr_	PF7	RV22	S2_2_9	S3_4_9	T265	VA1	VIB12	VIB43	VIB_18	VIB_93
030305-1_5	0.28957055	0.28957055	0.28957055	0.28957055	0.28959784	0.28957556	0.28957783	0.28959511	0.28965791	0.29001176	0.2900064	0.29118933	0.00103005	0.29295181	0.29531396	0.28840434	0.2897248	0.28490923	0.00096749	0.28845456	0.28852538
12B09	0.25545378	0.25545378	0.25545378	0.25545378	0.25548019	0.25545378	0.25545787	0.25547535	0.25547649	0.25573868	0.25593146	0.26201919	0.30659315	0.31721727	0.3169845	0.2542364	0.25567328	0.29782571	0.30581206	0.25438244	0.25439824
178_90	0.22315459	0.22315459	0.22315459	0.22315459	0.22315844	0.22315844	0.22317922	0.22320598	0.2232506	0.22364041	0.22357625	0.22665886	0.29085066	0.30197457	0.30112178	0.00072389	0.00172274	0.27898212	0.28859107	0.00036163	0.00027575
261_91	0.22315673	0.22315673	0.22315673	0.22315673	0.22316058	0.22316058	0.2231432	0.22316043	0.22325277	0.22364267	0.2235785	0.22668528	0.2908697	0.30194402	0.30109102	0.00068958	0.00168887	0.27895756	0.28857449	0.00043067	0.0004482
425	0.22346677	0.22346677	0.22346677	0.22346677	0.22347447	0.22346677	0.22345907	0.2234763	0.22358578	0.22397585	0.22391147	0.22698372	0.29122128	0.30229513	0.30144233	0.00106863	0.00206779	0.27928489	0.28894915	0.00070626	0.00062051
4299-E1-R1	0.28861761	0.28861761	0.28861761	0.28861761	0.28864484	0.28862259	0.28867599	0.28871781	0.28868009	0.28903293	0.28902822	0.29027581	0.07848848	0.29366874	0.29734116	0.28895262	0.29032537	0.23212584	0.07654436	0.28907073	0.28905359
4299	0.28858805	0.28858805	0.28858805	0.28858805	0.28861529	0.28859304	0.28864645	0.28868827	0.28865053	0.28900335	0.28899867	0.2902463	0.07847072	0.29363928	0.29731176	0.28892306	0.29029584	0.23209521	0.07652709	0.28904119	0.28902403
51_82_2	0.22314904	0.22314904	0.22314904	0.22314904	0.22315289	0.22315289	0.22316413	0.22318136	0.22324507	0.22363495	0.22357079	0.22667126	0.29085582	0.30198532	0.30113235	0.0007068	0.00170587	0.27902272	0.28865089	0.00034449	0.00012064
**531Ac**	0.22296144	0.22296144	0.22296144	0.22296144	0.22296529	0.22296144	0.22294992	0.22296715	0.22307653	0.22346629	0.22340224	0.22647525	0.29073795	0.30177688	0.30091866	0.00058615	0.00172348	0.27882404	0.28845124	0.00046519	0.00048272
**531Ad**	0.22327386	0.22327386	0.22327386	0.22327386	0.2232777	0.22327386	0.22326231	0.22327955	0.22338903	0.22377912	0.22371484	0.22677125	0.29105896	0.30203934	0.30118093	0.00093116	0.00206868	0.27916832	0.28876294	0.00080997	0.00082771
6018_1	0.22406303	0.22406303	0.22406303	0.22406303	0.22406689	0.22406689	0.22409128	0.22410851	0.22415934	0.22455002	0.22448522	0.22772397	0.29181912	0.30294097	0.30208782	0.00177617	0.00277505	0.2799917	0.28960464	0.00141282	0.00118953
601_90	0.2231375	0.2231375	0.2231375	0.2231375	0.22314135	0.22314135	0.22316413	0.22318136	0.22323352	0.22362338	0.22355923	0.2266368	0.29086163	0.30195247	0.30109955	0.00070676	0.00170578	0.27895638	0.28860139	0.00034449	0.00025855
775	0.22471034	0.22471034	0.22471034	0.22471034	0.22471813	0.22471034	0.22470256	0.22471988	0.22483024	0.22522319	0.22515765	0.22784657	0.29186808	0.30313788	0.30239163	0.00190549	0.00335971	0.28040452	0.28957784	0.0020948	0.0021134
87-9-116	0.22309164	0.22309164	0.22309164	0.22309164	0.22309548	0.22309164	0.22308965	0.22310688	0.22318381	0.22357366	0.22350953	0.2265975	0.29082901	0.30194923	0.30109621	0.0006551	0.00165443	0.2789278	0.28852765	0.00025842	0.0003103
87-9-117	0.22306844	0.22306844	0.22306844	0.22306844	0.22307228	0.22307228	0.22309693	0.22311416	0.22316443	0.22355417	0.22349007	0.22657273	0.29079223	0.30188322	0.30103043	0.00063771	0.0016366	0.27892544	0.28848744	0.00024109	0.00029298
90-11-286	0.29925724	0.29925724	0.29925724	0.29925724	0.29925032	0.2992624	0.29924693	0.29926416	0.29931222	0.29950149	0.29965523	0.30221939	0.30827829	0.2753326	0.27387558	0.30199155	0.30325283	0.30138502	0.30738137	0.30208513	0.30204328
90-11-287	0.22314904	0.22314904	0.22314904	0.22314904	0.22315673	0.22315289	0.22315089	0.22316813	0.22324507	0.22363495	0.22357079	0.22669309	0.29087358	0.30198532	0.30113235	0.0007068	0.00170607	0.27898732	0.28865588	0.00034455	5.17E-05
9014_8	0.22317626	0.22317626	0.22317626	0.22317626	0.22318396	0.22318011	0.22317811	0.22319535	0.22327234	0.22366241	0.2235982	0.22668735	0.29084288	0.30199479	0.30114146	0.00068986	0.00168957	0.27900419	0.28862505	0.00032746	0
91-8-178	0.22304935	0.22304935	0.22304935	0.22304935	0.2230532	0.2230532	0.22306447	0.22308169	0.22317212	0.22356188	0.22349778	0.22653666	0.29081805	0.30189882	0.30104599	0.00063773	0.00163672	0.27893991	0.28852465	3.44E-05	0.000293
96F	0.22281034	0.22281034	0.22281034	0.22281034	0.22281803	0.22281034	0.22280266	0.2228199	0.22292346	0.2233189	0.22326263	0.22596717	0.29007444	0.30091547	0.30073101	0.03792949	0.0392992	0.27598734	0.28783034	0.03802793	0.03806813
A023	0.22344569	0.22344569	0.22344569	0.22344569	0.22344954	0.22344954	0.2234607	0.22347793	0.22354178	0.22393183	0.22386748	0.22703625	0.29124421	0.30232278	0.30146995	0.00108591	0.00208466	0.27936448	0.28895644	0.00082666	0.00084454
ATCC-68554	0.22331369	0.22331369	0.22331369	0.22331369	0.22332138	0.22331369	0.22330599	0.22332322	0.22343268	0.22381495	0.22375838	0.22686541	0.29111249	0.30211439	0.3012615	0.00089635	0.0023437	0.27910154	0.28881864	0.00108533	0.00110322
ATCC_14181	0.28942319	0.28942319	0.28942319	0.28942319	0.28945048	0.28942319	0.28942547	0.28944275	0.28950053	0.28986661	0.28986134	0.29130157	0.00115457	0.29306441	0.29542391	0.28828626	0.28961201	0.28484964	0.00095036	0.28834154	0.28841231
Ba35-E2-2	0.22284492	0.22284492	0.22284492	0.22284492	0.22284877	0.22284877	0.22284295	0.2228602	0.22296773	0.22335761	0.2232936	0.22635263	0.29059541	0.30178464	0.300776	0.00017248	0.00182771	0.27863038	0.28836614	0.00056882	0.00058642
Ba35-E2-R1	0.22286611	0.22286611	0.22286611	0.22286611	0.22286996	0.22286996	0.22288128	0.22289853	0.22296213	0.22335209	0.22328807	0.22633929	0.29059373	0.30177811	0.30076926	0.00017252	0.00182784	0.2786247	0.28836413	0.00056883	0.00058652
Ba35-E2-R3	0.22287173	0.22287173	0.22287173	0.22287173	0.22287557	0.22287557	0.22288689	0.22290413	0.22296773	0.22335761	0.2232936	0.22636143	0.29058508	0.30178464	0.300776	0.00017248	0.00182746	0.27865336	0.28835618	0.00056871	0.0005864
Ba35-E2-R4	0.22287173	0.22287173	0.22287173	0.22287173	0.22287557	0.22287557	0.22288689	0.22290413	0.22296773	0.22335761	0.2232936	0.22636143	0.29058508	0.30178464	0.300776	0.00017248	0.00182746	0.27865336	0.28835618	0.00056871	0.0005864
Ba35	0.22286177	0.22286177	0.22286177	0.22286177	0.22286945	0.22286561	0.22286364	0.22288087	0.2229845	0.22337416	0.22331018	0.22638843	0.29056946	0.30166635	0.30077896	0.00017238	0.00182661	0.27868654	0.28834727	0.00056848	0.00058606
CNEVA_NB1	0.23638716	0.23638716	0.23638716	0.23638716	0.23639532	0.23639124	0.23639626	0.23641351	0.23648287	0.23682711	0.23690932	0.18759273	0.29495176	0.29949098	0.30233963	0.2452436	0.2464446	0.28013227	0.29396403	0.24527683	0.24535598
DSM_21597	0.28530752	0.28530752	0.28530752	0.28530752	0.28531244	0.28531244	0.28529772	0.28531497	0.28544551	0.28583027	0.28572415	0.2856921	0.27525693	0.25037098	0.25223018	0.28322542	0.28432354	0.28102708	0.27441756	0.28318034	0.28321564
FF-93	0.23231128	0.23231128	0.23231128	0.23231128	0.23231532	0.23231128	0.23229915	0.23231655	0.23239461	0.23273475	0.23279373	0.23507203	0.29267813	0.30574745	0.30857968	0.22517801	0.22640327	0.28183326	0.29149296	0.22518008	0.22523417
FS-144	0.25637163	0.25637163	0.25637163	0.25637163	0.25639364	0.25637163	0.25637118	0.2563887	0.25639453	0.25667099	0.25686354	0.26294681	0.3070991	0.31808867	0.31794378	0.25509954	0.25651717	0.29849776	0.30637685	0.2552409	0.25526173
HI610	0.29007066	0.29007066	0.29007066	0.29007066	0.29009295	0.29007568	0.29012868	0.29014595	0.29013359	0.29048772	0.29048203	0.2919437	0.00166895	0.29356483	0.29592648	0.28881129	0.29019716	0.28549142	0.00151994	0.28892459	0.28892961
HI618	0.288794	0.288794	0.288794	0.288794	0.28882127	0.288794	0.28879631	0.28881359	0.28887121	0.28923689	0.28923205	0.29030976	0.07882386	0.29388785	0.29757851	0.2892652	0.2905909	0.23218219	0.07688449	0.28933721	0.28939129
J360	0.28963193	0.28963193	0.28963193	0.28963193	0.28965422	0.28963694	0.2896342	0.28965148	0.28969475	0.29004861	0.29004322	0.29134919	0.00134962	0.29307991	0.29544905	0.28828564	0.28959104	0.28479784	0.00098463	0.28832098	0.2883967
JLL237	0.29921409	0.29921409	0.29921409	0.29921409	0.29921925	0.29920201	0.29916415	0.29918139	0.29932078	0.29983094	0.29968106	0.30572645	0.2966186	0.21554636	0.21012719	0.30387846	0.30535825	0.3006502	0.29750881	0.30422419	0.30424061
LMG12010	0.22361501	0.22361501	0.22361501	0.22361501	0.22361887	0.22361887	0.22364338	0.22366062	0.22371122	0.22410163	0.22403712	0.22700084	0.29133648	0.30245349	0.30151395	0.0011383	0.00213764	0.27944713	0.28904481	0.00087884	0.00089678
M3	0.22362949	0.22362949	0.22362949	0.22362949	0.22363721	0.22362949	0.22362178	0.22363903	0.22374871	0.22413942	0.22407484	0.22694411	0.291234	0.30221885	0.30138215	0.00110459	0.00210508	0.27929846	0.28895846	0.00074171	0.00065586
M93	0.28987386	0.28987386	0.28987386	0.28987386	0.28989614	0.28987386	0.2898711	0.28988838	0.28995627	0.29032258	0.290317	0.29159108	0.00131371	0.29334117	0.29570506	0.28863377	0.28995402	0.28522885	0.00131281	0.28868384	0.28875979
MHK3	0.26562096	0.26562096	0.26562096	0.26562096	0.26564278	0.26562096	0.26560723	0.26562446	0.26569315	0.26590293	0.26603448	0.27085955	0.29757908	0.29527552	0.29532527	0.26770594	0.26889061	0.27752413	0.29673596	0.26778574	0.26790026
MVAV6203	0.2231952	0.2231952	0.2231952	0.2231952	0.2232029	0.2231952	0.22318751	0.22320474	0.22331034	0.223706	0.2236495	0.22673056	0.2909895	0.3020494	0.30118592	0.0008276	0.00182715	0.27902593	0.28872801	0.00046525	0.00037932
NB10	0.22314805	0.22314805	0.22314805	0.22314805	0.22315575	0.2231519	0.22314035	0.2231576	0.22327098	0.22365354	0.22359702	0.22661722	0.29093365	0.30211658	0.30126281	0.00072474	0.00172509	0.27903072	0.288686	0.00032766	0.00037962
NCTC12159	0.28562303	0.28562303	0.28562303	0.28562303	0.28562796	0.28562303	0.2856107	0.28562797	0.28573156	0.28612907	0.28602269	0.28603981	0.27557192	0.25066036	0.25252473	0.28333765	0.28444122	0.2812749	0.27473136	0.28329737	0.28333276
NN_sinnomb	0.22313736	0.22313736	0.22313736	0.22313736	0.22314121	0.22314121	0.22316584	0.22318306	0.22323337	0.22362316	0.22355902	0.22664163	0.2908329	0.30195734	0.30110454	0.00070665	0.00170551	0.27896484	0.2885738	0.00034441	0.00025851
PF4-E1-1	1.72E-05	1.72E-05	1.72E-05	1.72E-05	3.45E-05	1.72E-05	0	1.72E-05	0.0001034	0.00048288	0.00049978	0.22589147	0.29161932	0.29809233	0.2992107	0.22309005	0.22433047	0.27968329	0.28959596	0.22313907	0.22317811
PF4-E1-2	3.45E-05	3.45E-05	3.45E-05	3.45E-05	5.17E-05	3.45E-05	1.72E-05	0	0.00012063	0.00050012	0.00051702	0.2259087	0.29163707	0.29810957	0.29922793	0.22310729	0.22434771	0.27970057	0.28961324	0.2231563	0.22319535
PF4-E1-3	3.45E-05	3.45E-05	3.45E-05	3.45E-05	5.17E-05	3.45E-05	1.72E-05	3.45E-05	0.00012069	0.00050035	0.00051726	0.22587595	0.29156378	0.29816213	0.29926378	0.22309046	0.22433145	0.27962364	0.28954099	0.2231395	0.22317856
PF4-E1-4	1.72E-05	1.72E-05	1.72E-05	1.72E-05	3.45E-05	1.72E-05	0	1.72E-05	0.00010339	0.00048287	0.00049978	0.22590481	0.29161932	0.29810443	0.2992278	0.22310345	0.22434384	0.27969574	0.28959096	0.22315245	0.2231915
PF4-E1-5	1.72E-05	1.72E-05	1.72E-05	1.72E-05	3.45E-05	1.72E-05	0	1.72E-05	0.0001034	0.00048288	0.00049978	0.22590647	0.29161932	0.29809233	0.2992107	0.22309005	0.22433047	0.27968329	0.28959596	0.22313907	0.22317811
PF4-E2-2	0	0	0	0	1.72E-05	0	1.72E-05	3.45E-05	8.62E-05	0.00050014	0.00048254	0.2258637	0.2916171	0.29813707	0.29925548	0.22309775	0.22430406	0.27970952	0.2895937	0.22311059	0.22317626
PF4-E2-3	0	0	0	0	1.72E-05	0	1.72E-05	3.45E-05	8.62E-05	0.00050014	0.00048254	0.2258637	0.2916171	0.29813707	0.29925548	0.22309775	0.22430406	0.27970952	0.2895937	0.22311059	0.22317626
PF4-E2-5	0	0	0	0	1.72E-05	0	1.72E-05	3.45E-05	8.62E-05	0.00050014	0.00048254	0.2258637	0.2916171	0.29813707	0.29925548	0.22309775	0.22430406	0.27970952	0.2895937	0.22311059	0.22317626
PF4-E2-6	0	0	0	0	1.72E-05	0	1.72E-05	3.45E-05	8.62E-05	0.00050014	0.00048254	0.2258637	0.2916171	0.29813707	0.29925548	0.22309775	0.22430406	0.27970952	0.2895937	0.22311059	0.22317626
PF4-E2-R1	1.72E-05	1.72E-05	1.72E-05	1.72E-05	0	1.72E-05	3.45E-05	5.17E-05	0.0001034	0.0005174	0.00049979	0.22586759	0.29164521	0.29814735	0.2992658	0.22310544	0.22430793	0.27973165	0.28961598	0.22311443	0.22318396
PF4-E2-R4	0	0	0	0	1.72E-05	0	1.72E-05	3.45E-05	8.62E-05	0.00050014	0.00048254	0.22586759	0.2916171	0.29812498	0.2992434	0.22309775	0.22430793	0.27971435	0.2895987	0.22311443	0.22318011
PF4	1.72E-05	1.72E-05	1.72E-05	1.72E-05	3.45E-05	1.72E-05	0	1.72E-05	0.0001034	0.00048288	0.00049978	0.22589147	0.29161932	0.29809233	0.2992107	0.22309005	0.22433047	0.27968329	0.28959596	0.22313907	0.22317811
PF430-3	3.45E-05	3.45E-05	3.45E-05	3.45E-05	5.17E-05	3.45E-05	1.72E-05	0	0.00012063	0.00050012	0.00051702	0.2259087	0.29163707	0.29810957	0.29922793	0.22310729	0.22434771	0.27970057	0.28961324	0.2231563	0.22319535
PF4_substr_	8.62E-05	8.62E-05	8.62E-05	8.62E-05	0.0001034	8.62E-05	0.0001034	0.00012063	0	0.00043115	0.00043089	0.22596046	0.29167703	0.29819178	0.29927083	0.22321675	0.22440042	0.27984642	0.28965648	0.22320659	0.22327234
PF4_substr_	0.00050014	0.00050014	0.00050014	0.00050014	0.0005174	0.00050014	0.00048288	0.00050012	0.00043115	0	0.00032773	0.22635228	0.29203178	0.29844747	0.29945316	0.22360677	0.22479122	0.28020561	0.29002248	0.22359638	0.22366241
PF4_substr_	0.00048254	0.00048254	0.00048254	0.00048254	0.00049979	0.00048254	0.00049978	0.00051702	0.00043089	0.00032773	0	0.22637249	0.29198416	0.29855712	0.2996828	0.2235426	0.22472627	0.28024145	0.29001711	0.22353225	0.2235982
PF7	0.2258637	0.2258637	0.2258637	0.2258637	0.22586759	0.22586759	0.22589147	0.2259087	0.22596046	0.22635228	0.22637249	0	0.29292804	0.30179381	0.30144398	0.22653058	0.22783828	0.28046124	0.29131043	0.22659776	0.22668735
RV22	0.2916171	0.2916171	0.2916171	0.2916171	0.29164521	0.2916171	0.29161932	0.29163707	0.29167703	0.29203178	0.29198416	0.29292804	0	0.29469311	0.29747869	0.29076275	0.2921492	0.28627682	0.00198828	0.29084323	0.29084288
S2_2_9	0.29813707	0.29813707	0.29813707	0.29813707	0.29814735	0.29812498	0.29809233	0.29810957	0.29819178	0.29844747	0.29855712	0.30179381	0.29469311	0	0.05511906	0.30185686	0.30315252	0.29944841	0.2931788	0.30193328	0.30199479
S3_4_9	0.29925548	0.29925548	0.29925548	0.29925548	0.2992658	0.2992434	0.2992107	0.29922793	0.29927083	0.29945316	0.2996828	0.30144398	0.29747869	0.05511906	0	0.30100352	0.30226505	0.29858385	0.29554208	0.30108046	0.30114146
T265	0.22309775	0.22309775	0.22309775	0.22309775	0.22310544	0.22309775	0.22309005	0.22310729	0.22321675	0.22360677	0.2235426	0.22653058	0.29076275	0.30185686	0.30100352	0	0.00193107	0.27881388	0.28851405	0.00067221	0.00068986
VA1	0.22430406	0.22430406	0.22430406	0.22430406	0.22430793	0.22430793	0.22433047	0.22434771	0.22440042	0.22479122	0.22472627	0.22783828	0.2921492	0.30315252	0.30226505	0.00193107	0	0.28013835	0.28981877	0.00167112	0.00168957
VIB12	0.27970952	0.27970952	0.27970952	0.27970952	0.27973165	0.27971435	0.27968329	0.27970057	0.27984642	0.28020561	0.28024145	0.28046124	0.28627682	0.29944841	0.29858385	0.27881388	0.28013835	0	0.28506803	0.27897448	0.27900419
VIB43	0.2895937	0.2895937	0.2895937	0.2895937	0.28961598	0.2895987	0.28959596	0.28961324	0.28965648	0.29002248	0.29001711	0.29131043	0.00198828	0.2931788	0.29554208	0.28851405	0.28981877	0.28506803	0	0.28854922	0.28862505
VIB_18	0.22311059	0.22311059	0.22311059	0.22311059	0.22311443	0.22311443	0.22313907	0.2231563	0.22320659	0.22359638	0.22353225	0.22659776	0.29084323	0.30193328	0.30108046	0.00067221	0.00167112	0.27897448	0.28854922	0	0.00032746
VIB_93	0.22317626	0.22317626	0.22317626	0.22317626	0.22318396	0.22318011	0.22317811	0.22319535	0.22327234	0.22366241	0.2235982	0.22668735	0.29084288	0.30199479	0.30114146	0.00068986	0.00168957	0.27900419	0.28862505	0.00032746	0

Supplementary Table S2

Reference sequence: 531Ac

Target: 531Ad

Mapping: 99,7%

Predicted mutations				
**position**	**mutation**	**annotation**	**gene**	**description**
723	A→T	intergenic (+228/+198)	*531AC_00001 → / ← 531AC_00002*	hypothetical protein/hypothetical protein
36,036	A→T	intergenic (+9/+19)	*531AC_00035 → / ← yqaB*	hypothetical protein/Fructose-1-phosphate phosphatase YqaB
352,218	A→G	intergenic (+17/-17)	*531AC_00327 → / → 531AC_00328*	tRNA-Met/tRNA-Gly
485,760	+GA	intergenic (-50/+6)	*531AC_00457 ← / ← 531AC_00458*	tRNA-Thr/tRNA-Phe
485,762	T→A	intergenic (-52/+4)	*531AC_00457 ← / ← 531AC_00458*	tRNA-Thr/tRNA-Phe
725,655	T→C	intergenic (+12/+308)	*yadH → / ← 531AC_00689*	Inner membrane transport permease YadH/hypothetical protein
725,661	C→A	intergenic (+18/+302)	*yadH → / ← 531AC_00689*	Inner membrane transport permease YadH/hypothetical protein
894,336	C→A	intergenic (+367/-281)	*clpB → / → 531AC_00833*	Chaperone protein ClpB/tRNA-Glu
916,537	T→C	intergenic (-160/-127)	*531AC_00851 ← / → 531AC_00852*	hypothetical protein/tRNA-Trp
1,538,578	$(T)_{8\rightarrow9}$	intergenic (+68/-128)	*lon_1 → / → hupB*	Lon protease/DNA-binding protein HU-beta
2,199,419	A→C	*372S (TAG→TCG)	*531AC_01990 →*	IS66 family transposase ISVa5
2,388,122	A→C	Q349P (CAG→CCG)	*tyrP →*	Tyrosine-specific transport protein
2,419,430	Δ1 bp	intergenic (+99/+172)	*531AC_02194 → / ← nadA*	hypothetical protein/Quinolinate synthase A
2,449,349	G→A	P349L (CCT→CTT)	*531AC_02227 ←*	hypothetical protein
2,553,829	Δ3 bp	intergenic (-31/+490)	*oppA ← / ← rssB_5*	Periplasmic oligopeptide-binding protein/Regulator of RpoS
3,708,771	G→C	intergenic (-580/+671)	*531AC_03417 ← / ← rsgA_2*	hypothetical protein/Small ribosomal subunit biogenesis GTPase RsgA
3,785,999	+43 bp	intergenic (-235/-160)	*531AC_03494 ← / → 531AC_03495*	hypothetical protein/IS5 family transposase ISVa2
3,786,709	G→C	G184A (GGC→GCC)	*531AC_03495 →*	IS5 family transposase ISVa2
3,786,712	Δ1 bp	coding (554/582 nt)	*531AC_03495 →*	IS5 family transposase ISVa2
4,010,366	Δ1 bp	intergenic (-189/-97)	*531AC_03678 ← / → 531AC_03679*	hypothetical protein/hypothetical protein
4,010,371	C→G	intergenic (-194/-92)	*531AC_03678 ← / → 531AC_03679*	hypothetical protein/hypothetical protein
4,063,818	Δ1 bp	intergenic (+140/-461)	*531AC_03725 → / → 531AC_03726*	hypothetical protein/hypothetical protein
4,103,461	T→G	intergenic (-1036/+785)	*531AC_03776 ← / ← 531AC_03777*	hypothetical protein/hypothetical protein
